# YTHDC1 drives senescence evasion in ovarian cancer through m6A-mediated TERT stabilization

**DOI:** 10.1038/s41419-026-08896-7

**Published:** 2026-05-30

**Authors:** Fen Wang, Zhengyang Zhao, Zhuanghua Li, Xiaolei Chen, Weihong Lu, Shuying Yao, Jiameng Xi, Yanshuo Sun, Huirou Luo, Qingyuan Feng, Shuguang Lv, Piao Xu, Lingjia Shen, Wenqing Zhang, Yan-yan Zhan, Liangxi Xie, Tianhui Hu, Xiaoting Hong

**Affiliations:** 1 Xiamen Key Laboratory for Tumor Metastasis, Cancer Research Center, School of Medicine, Xiamen University, Xiamen, China; 2 Dongguan Institute of Clinical Cancer Research, Dongguan Key Laboratory of Precision Diagnosis and Treatment for Tumors, The Tenth Affiliated Hospital of Southern Medical University (Dongguan People’s Hospital), Dongguan, China; 3 Department of Joint Surgery and Sports Medicine, Zhongshan Hospital Affiliated to Xiamen University, Xiamen, China; 4 Department of Gynecology, Zhongshan Hospital (Xiamen) of Fudan University, Xiamen Clinical Research Center for Cancer Therapy, Clinical Research Center for Precision Medicine of Abdominal Tumor of Fujian Province, Xiamen, China; 5 Department of Radiation Oncology, Xiang’an Hospital of Xiamen University, Cancer Research Center, School of Medicine, Xiamen University, Xiamen, China; 6 Shenzhen Research Institute of Xiamen University, Shenzhen, China

**Keywords:** Ovarian cancer, Senescence

## Abstract

N6-methyladenosine (m6A) represents the most abundant internal RNA modification, and a key regulator of gene expression, yet its role in determining cell fate decisions such as senescence remains largely unexplored. Here, we identify the nuclear m6A reader YTHDC1 as a critical regulator of telomere homeostasis and senescence evasion in ovarian cancer. YTHDC1 expression was markedly elevated in advanced-stage tumors and correlated with poor patient survival. Functional investigation demonstrated that YTHDC1 ablation induced profound proliferative arrest and established a canonical senescence landscape. Mechanistic dissection revealed that YTHDC1 directly recognizes m6A-modified TERT transcripts, thereby stabilizing telomerase mRNA and maintaining telomere homeostasis. YTHDC1 knockdown triggered telomerase dysfunction and accelerated telomere shortening. Genetic restoration of TERT effectively reversed both the proliferative defects and senescence phenotypes induced by YTHDC1 deficiency. Significantly, YTHDC1-depleted senescent cells displayed enhanced sensitivity to the senolytic agent, ABT-263. Collectively, these findings uncover a previously unrecognized epitranscriptomic–telomerase axis that dictates senescence escape, establishing YTHDC1 as a central node linking RNA modification to telomere maintenance, cellular senescence, and tumor progression.

**Figure:**
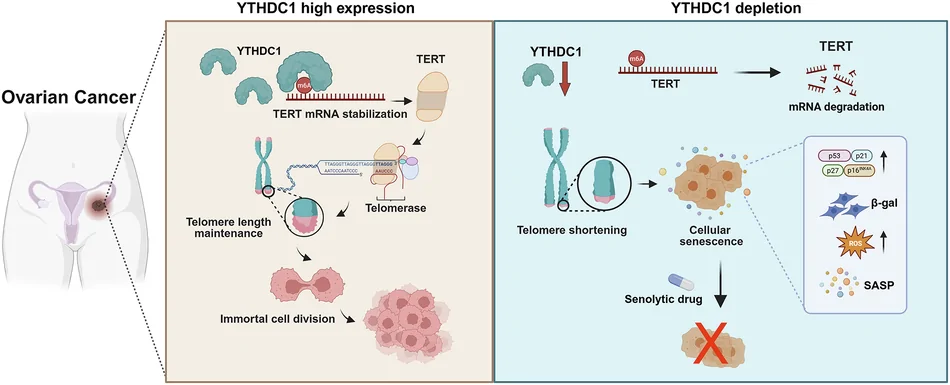

## Introduction

N6-methyladenosine (m6A), the most prevalent internal mRNA modification in eukaryotes [1], is dynamically regulated by “writers”, “erasers”, and “readers”, which collectively fine-tune post-transcriptional gene expression [2–4]. Aberrant m6A metabolism is increasingly implicated in tumorigenesis [4, 5]. Yet its role in cellular senescence—a potent tumor-suppressive barrier—remains poorly defined.

Cellular senescence is characterized by irreversible cell cycle arrest and the adoption of a complex senescence-associated secretory phenotype (SASP), which functions as a potent intrinsic tumor suppressor mechanism triggered by various cellular stresses, such as oncogene activation, DNA damage, and telomere attrition [6]. Consequently, nascent cancer cells must evolve strategies to circumvent this barrier and achieve sustained proliferation. A central mechanism enabling senescence bypass is the reactivation of telomerase, the enzyme complex responsible for maintaining telomere length, primarily through upregulation of its catalytic subunit, telomerase reverse transcriptase (TERT) [7]. Although transcriptional and epigenetic mechanisms governing TERT expression are well-documented [8], whether post-transcriptional regulation via m6A modification influences telomerase activity and facilitates senescence evasion remains a critical unanswered question.

Ovarian cancer, characterized by high mortality and chemoresistance, underscores the need for novel therapeutic insights [9–11]. Here, we identified the nuclear m6A reader, YTHDC1, as a pivotal regulator of senescence evasion in ovarian cancer. We found that YTHDC1 was significantly upregulated in ovarian tumor tissues, and its expression levels correlated strongly with advanced disease stage and poor patient survival. Functionally, depletion of YTHDC1 robustly induced cellular senescence, which was characterized by growth arrest and canonical senescence markers. Mechanistically, we demonstrated that YTHDC1 directly binds to m6A-modified TERT mRNA, thereby enhancing its stability and sustaining telomerase activity. Consequently, the loss of YTHDC1 compromises telomerase function, leading to accelerated telomere shortening and senescence induction. Significantly, YTHDC1-depleted senescent cells exhibited heightened sensitivity to the senolytic agent ABT-263, highlighting their potential therapeutic vulnerability.

Collectively, our findings unveil an epitranscriptomic pathway through which YTHDC1 governs telomerase activity and senescence escape, positioning it as a prognostic biomarker and a promising therapeutic target for senescence-directed therapy in ovarian cancer.

## Material and methods

### Cell lines and cell culture

A2780, ES-2, SKOV3, OVCAR3, IOSE-80, and HEK293T cells were obtained from Meisen Cell Technology (Zhejiang, China). All cell lines were authenticated by STR profiling and routinely tested for mycoplasma contamination, and were confirmed to be mycoplasma-negative. A2780, IOSE-80, and HEK293T cells were cultured in DMEM; ES-2 and SKOV3 in McCoy’s 5A; and OVCAR3 in RPMI-1640 supplemented with 20% FBS and 0.01 mg/mL insulin. All media contained 10% FBS, 100 U/mL penicillin, and 100 μg/mL streptomycin.

### Plasmid construction

YTHDC1 and TERT cDNAs were cloned into pHIV-neo, pcDNA3.1, or pLVX-puro. Point mutations (W377A, W428A) were generated using site-directed mutagenesis. shYTHDC1 oligonucleotides were inserted into pLKO.1-puro. All constructs were verified by sequencing; primer sequences are listed in Table S1.

### Western blotting

Whole-cell lysates were prepared, and protein concentrations were determined using a BCA assay (A65453, Thermo Fisher Scientific, Waltham, MA, USA) according to the manufacturer’s instructions. Equal amounts of protein were resolved by SDS–PAGE and transferred onto PVDF membranes. Membranes were blocked with 5% skim milk and incubated with the primary antibodies listed in Table S2, followed by HRP-conjugated secondary antibodies. Immunoreactive bands were detected using an ECL kit (P40100, NCM Biotech, Suzhou, China). Uncropped, full-length images of all immunoblots are provided in the Supplemental Material.

### RNA immunoprecipitation (RIP)

RIP was performed as described [12]. Briefly, cells were lysed in RIP lysis buffer on ice. Protein A/G agarose beads (sc-2003, Santa Cruz, Dallas, Texas, USA) were pre-washed with NT2 buffer and incubated with anti-YTHDC1 or control IgG antibodies. Cell lysates were then added to the antibody-conjugated beads and incubated at 4 °C overnight. Beads were washed with NT2 buffer, and RNA was extracted using TRIzol. Purified RNA was reverse-transcribed into cDNA, and the enrichment of target RNA was quantified by RT–qPCR.

### Telomerase repeated amplification protocol (TRAP)

Telomerase activity was measured using a modified TRAP assay [13]. Cells were lysed in ice-cold NP-40 buffer. TRAP reactions were prepared in 50 µL volumes containing TRAP buffer, dNTPs, primer mix (comprising the Cy5-labeled TS substrate, internal standard control TSNT, reverse primers NT, and ACX), 0.4 mg/mL BSA, Taq DNA polymerase, and cell lysate. After telomerase extension at 25 °C for 40 min and heat inactivation at 95 °C for 5 min, PCR amplification was performed for 29 cycles (95 °C 30 s, 52 °C 30 s, 72 °C 45 s). Products were resolved on non-denaturing polyacrylamide gels and visualized with a Typhoon FLA 9500 scanner. Primer sequences are listed in Table S3.

### Statistical analysis

All statistical analyses were performed using GraphPad Prism version 9. Data are presented as mean ± standard deviation (SD). For comparisons between two groups, an unpaired two-tailed Student’s *t* test was used for parametric data, and the Mann–Whitney U test was used for non-parametric data. For comparisons among three or more groups, one-way ANOVA with Tukey’s test was used for parametric data, and the Kruskal–Wallis test with Dunn’s test was used for non-parametric data. Time-course data were analyzed by two-way ANOVA where indicated. Significance: Statistical significance is indicated as follows: * *P* < 0.05; ** *P* < 0.01; and *** *P* < 0.001.

Supplementary Information contains further detailed information on all methods employed.

## Results

### YTHDC1 expression is elevated in ovarian cancer and correlates with advanced disease and poor patient survival

**Fig. 1:**
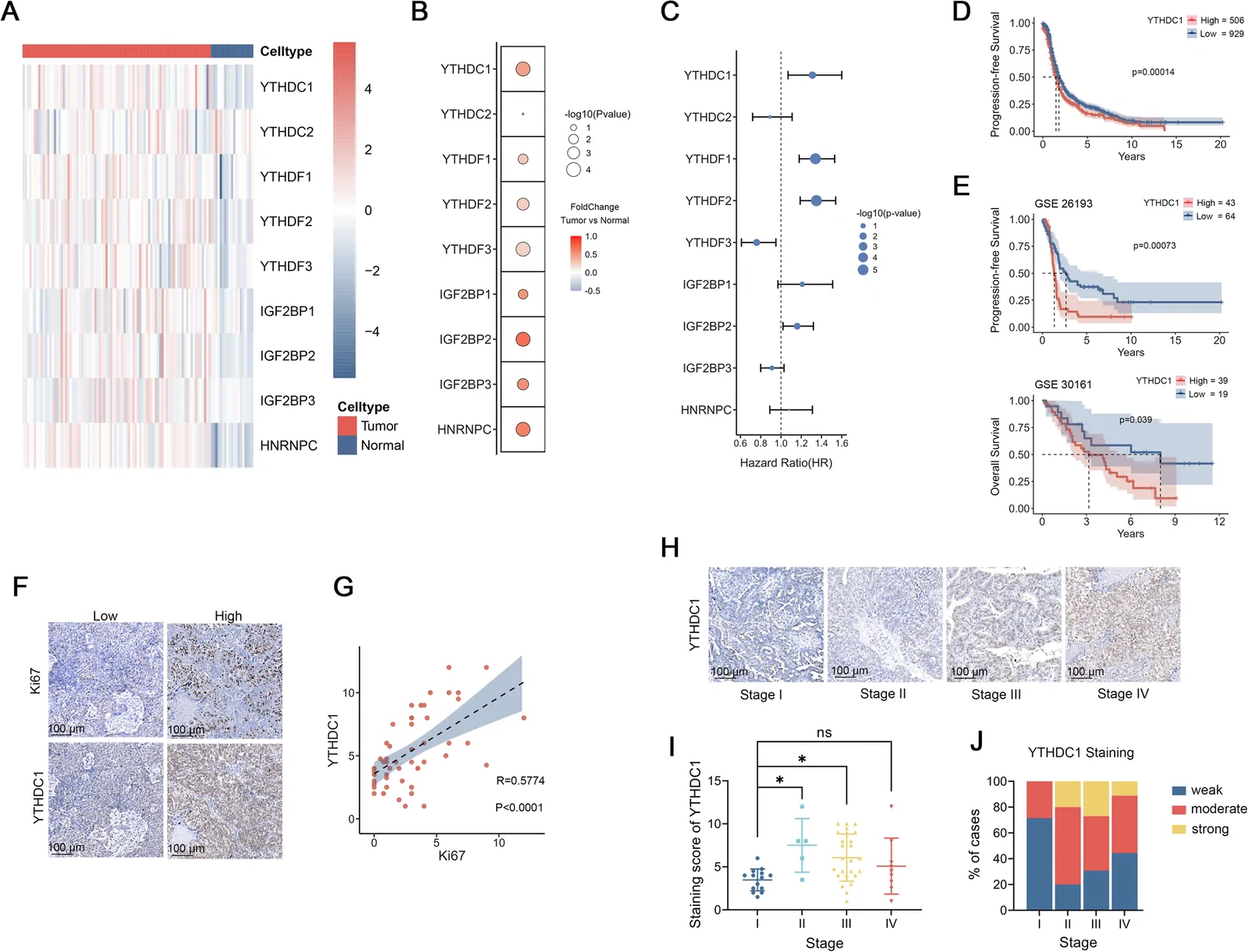
YTHDC1 is upregulated in tumors and correlates with poor prognosis. **A** Heatmaps showing the protein expression levels of m6A readers in ovarian tumor and normal tissues from CPTAC. **B** Dot plot comparing the differential expression of m6A readers between ovarian tumor and normal tissues from CPTAC. **C** Forest plot showing the Hazard Ratios (HRs) for nine m6A readers in ovarian cancer patients from KM plot database (www.kmplot.com). Kaplan-Meier survival analyses showing that high YTHDC1 expression correlates with poorer progression-free survival and overall survival across different cohorts (**D**) ovarian cancer datasets retrieved from KM plot database (**E**) GEO datasets (GSE26193 and GSE30161). *P* values were determined by log-rank test. **F** Representative immunohistochemistry (IHC) images of Ki67 and YTHDC1 staining in tumor tissues of 54 patients with ovarian cancer. **G** Correlation between YTHDC1 and Ki67 expression levels. R value and *P* value were determined by Spearman correlation. **H** Representative IHC images showing YTHDC1 staining in tumors across clinical stages I–IV. **I** Quantification of YTHDC1 staining intensity across tumor stages. **J** Proportion of cases with weak, moderate, and strong YTHDC1 staining in each tumor stage. Data are presented as mean ± SD. **B** Mann–Whitney U test; (**I**) Kruskal–Wallis test with Dunn’s test. ns, not significant; * *P* < 0.05.

To investigate m6A RNA methylation in ovarian cancer, we analyzed proteomic data from the Clinical Proteomic Tumor Analysis Consortium (CPTAC) via the cProSite platform [14], comprising 19 normal ovarian tissue samples and 84 ovarian tumor samples. We observed widespread upregulation of m6A readers in tumor tissues, particularly YTHDC1, IGF2BP1, and HNRNPC (Fig. 1A, B). Survival analysis using the KMplot database confirmed YTHDF1 and YTHDF2 as poor prognostic markers, consistent with previous reports [15, 16], and further identified YTHDC1 as a strong negative prognostic factor with a hazard ratio (HR) greater than 1(Fig. 1C). This finding was validated in TCGA and GEO datasets, where high YTHDC1 expression correlated with significantly worse progression-free and overall survival (Fig. 1D, E). Immunohistochemistry in 54 epithelial ovarian cancer patients revealed a positive correlation between YTHDC1 and the proliferation marker Ki67 (Fig. 1F, G). Moreover, YTHDC1 expression was elevated in advanced-stage (II/III) compared to early-stage (I) tumors (Fig. 1H, I), with the proportion of strongly positive cases increasing with disease progression (Fig. 1J).

Overall, our results indicate that YTHDC1 is significantly upregulated in ovarian cancer tissues and serves as a strong negative prognostic marker associated with poor patient outcomes and advanced tumor stages.

### Knockdown of YTHDC1 inhibits the proliferation of ovarian cancer cells

**Fig. 2:**
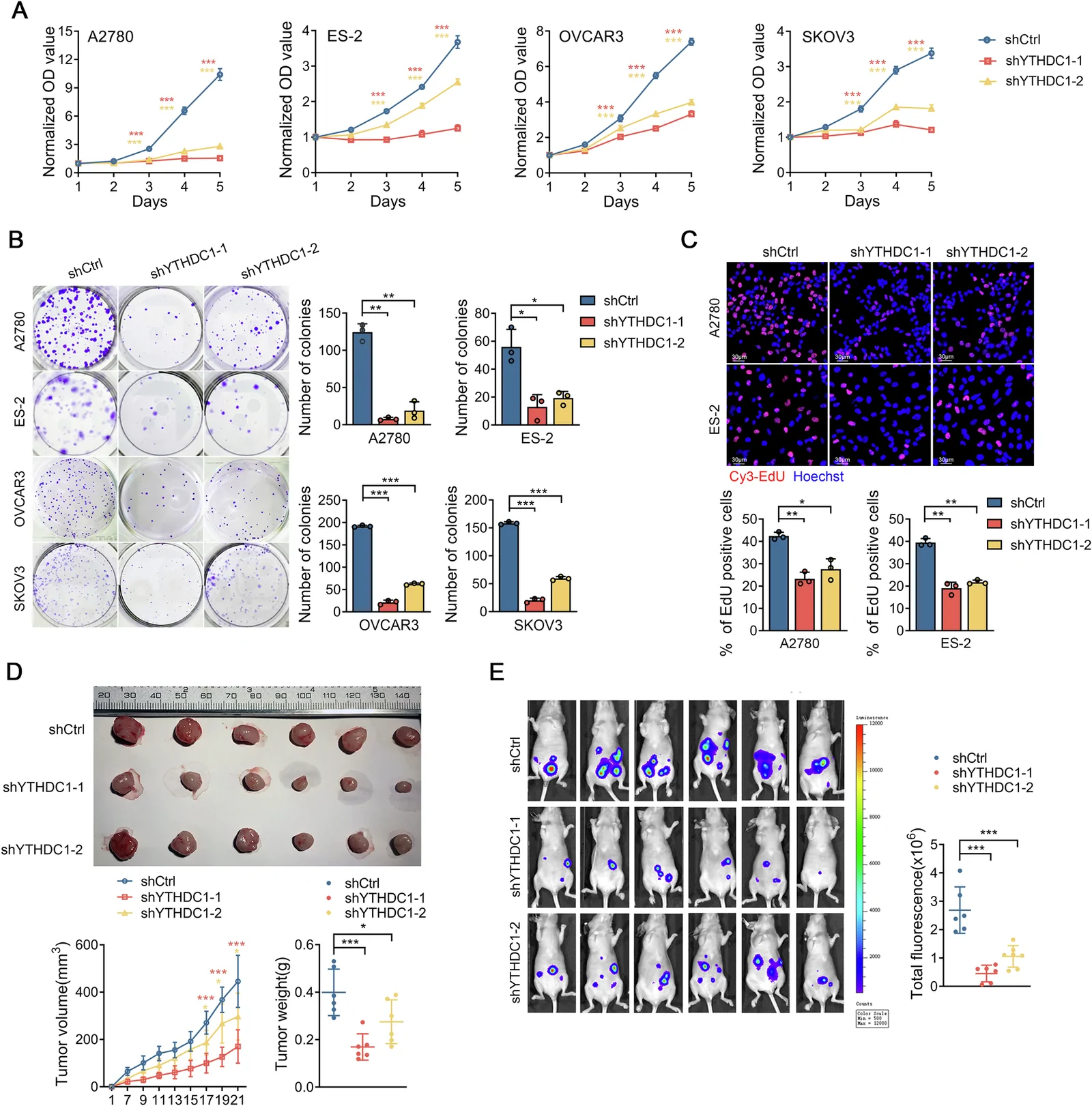
YTHDC1 knockdown inhibits the proliferation of ovarian cancer cells. **A** Cell viability assessed by MTT assays, *n* = 5. **B** Colony formation assays and corresponding quantitative analysis, *n* = 3. **C** Representative EdU staining images (top) and quantification of EdU-positive cells (bottom) in A2780 and ES-2 cells, *n* = 3. **D** Subcutaneous xenograft tumor model using OVCAR3 cells in nude mice: representative tumor images, tumor growth curves, and final tumor weights, *n* = 6 mice per group. **E** Representative bioluminescence imaging (BLI) of mice transplanted with luciferase-labeled A2780 cells (left), and quantification of total BLI signals in the abdominal region (right). *n* = 6 mice per group. Data are presented as mean ± SD. For time-course curves (MTT and tumor growth), two-way ANOVA with Dunnett’s test was used; for endpoint comparisons, two-sided unpaired Student’s *t* test (two groups) or one-way ANOVA with Tukey’s test ( ≥ 3 groups) was used. ns, not significant; * *P* < 0.05; ** *P* < 0.01; *** *P* < 0.001.

To define the functional role of YTHDC1 in ovarian cancer growth, we first confirmed its consistent upregulation in multiple ovarian cancer cell lines (A2780, ES-2, SKOV3, and OVCAR3) versus the normal ovarian epithelial cell line IOSE-80 (Fig. S1A). Stable knockdown of YTHDC1 (Fig. S1B) markedly suppressed proliferative capacity in vitro, as shown by impaired cell growth (Fig. 2A), colony formation (Fig. 2B), and EdU incorporation (Fig. 2C). In vivo, YTHDC1 knockdown in OVCAR3 xenografts led to significantly reduced tumor growth and weight (Fig. 2D). Furthermore, in an intraperitoneal metastasis model using luciferase-labeled A2780 cells, YTHDC1 depletion strongly attenuated peritoneal tumor burden (Fig. 2E). These findings demonstrated that YTHDC1 plays a crucial role in promoting ovarian cancer cell proliferation and tumor growth both in vitro and in vivo.

### Knockdown of YTHDC1 promotes senescence in ovarian cancer cells

**Fig. 3:**
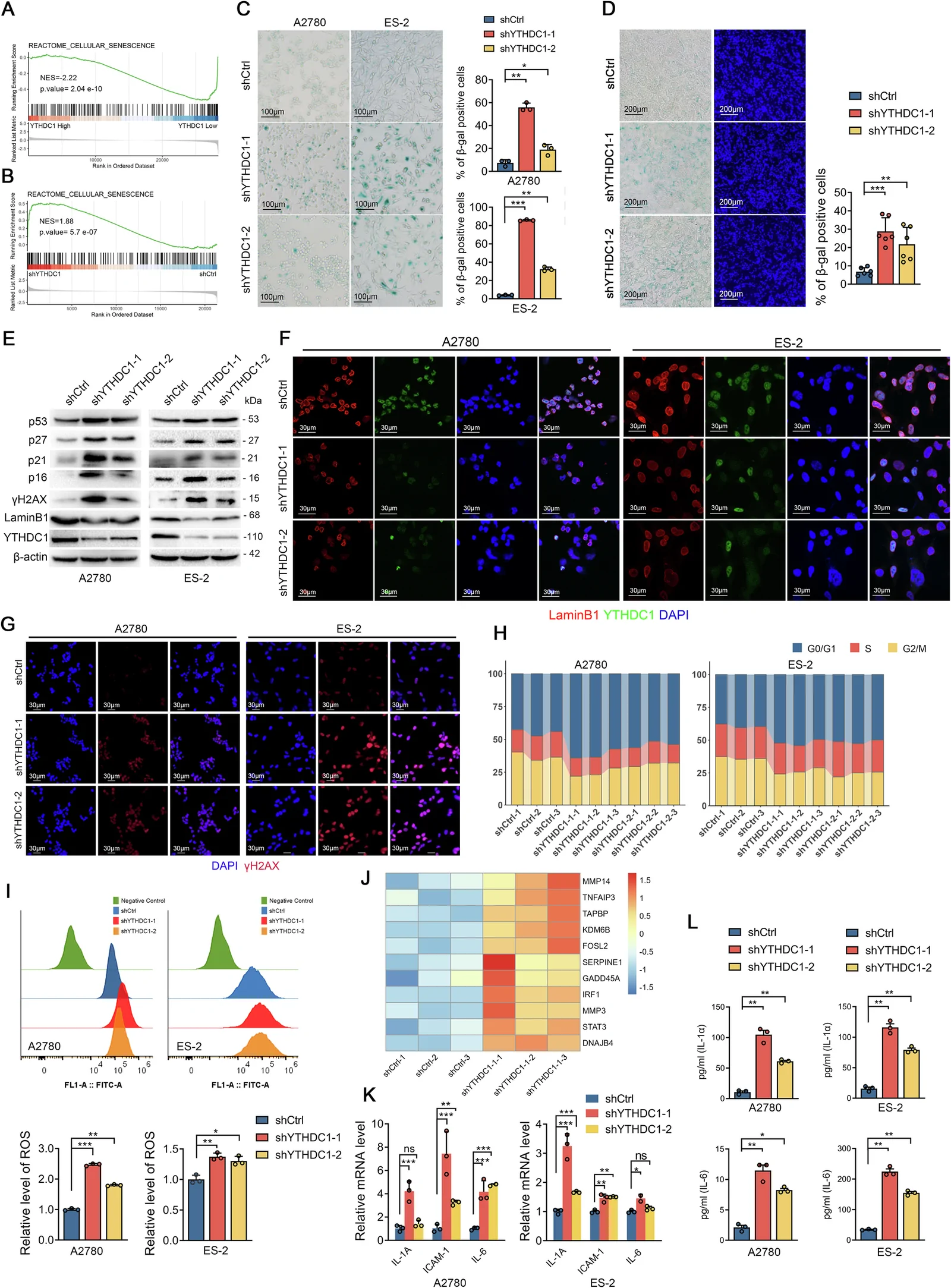
YTHDC1 knockdown induces cellular senescence in ovarian cancer cells. **A** Gene set enrichment analysis (GSEA) of TCGA ovarian cancer samples reveals significant enrichment of the cellular senescence pathway in tumors with low versus high YTHDC1 expression. **B** GSEA of RNA-Seq data from YTHDC1-knockdown A2780 cells confirms significant enrichment of the cellular senescence pathway. **C** Representative images (left) and quantification (right) of β-galactosidase staining in A2780 and ES-2 cells, *n* = 3. **D** Representative images (left) and quantification (right) of β-galactosidase staining in subcutaneous xenograft tumor sections. **E** Western blot analysis showing expression of senescence markers (p53, p21, p27, p16, γH2AX), nuclear envelope marker (LaminB1), and YTHDC1 in A2780 and ES-2 cells. **F** Immunofluorescence staining showing LaminB1 (red) and YTHDC1 (green) expression in A2780 and ES-2 cells. Nuclei were counterstained with DAPI (blue). **G** Representative immunofluorescence images of γH2AX (red) expression in the indicated cell lines. Nuclei were counterstained with DAPI (blue). **H** Cell cycle distribution analysis by flow cytometry in A2780 and ES-2 cells, showing percentage of cells in G0/G1, S, and G2/M phases. **I** Flow cytometry analysis of reactive oxygen species (ROS) production in A2780 and ES-2 cells (left) and quantification (right), *n* = 3. **J** Heatmap showing expression of senescence-associated secretory phenotype (SASP) genes in control and YTHDC1-knockdown cells based on RNA-seq analysis. **K** qRT-PCR analysis of proinflammatory cytokines (IL-1A, ICAM-1, IL-6) in A2780 and ES-2 cells, *n* = 3. **L** ELISA analysis of IL-1α and IL-6 in conditioned medium, *n* = 3. Data are presented as the mean ± SD. The difference significance was analyzed by one-way ANOVA with Tukey’s test. ns, not significant; * *P* < 0.05; ** *P* < 0.01; *** *P* < 0.001.

To uncover the mechanism by which YTHDC1 loss inhibits proliferation, we performed gene set enrichment analysis (GSEA) on TCGA data, which revealed significant enrichment of the senescence pathway in YTHDC1-low ovarian tumors (Fig. 3A). This finding was corroborated by RNA-Seq of YTHDC1-knockdown A2780 cells (Fig. 3B). We then systematically characterized multiple hallmarks of cellular senescence in YTHDC1-deficient cells. We observed a marked increase in β-galactosidase activity both in vitro and in xenograft models of YTHDC1-knockdown cells (Fig. 3C, D). Western blot and immunofluorescence analyses confirmed senescence-associated alterations, including upregulation of cell-cycle inhibitors (p53, p21, p16, p27) and the DNA damage marker γH2AX, together with loss of Lamin B1—indicative of impaired nuclear envelope integrity (Figs. 3E–G and S1C). Furthermore, YTHDC1 knockdown induced G1 cell cycle arrest (Figs. 3H and S1D), elevated ROS levels (Fig. 3I), and promoted the secretion of the SASP factors (Fig. 3J–L). Collectively, these findings demonstrate that YTHDC1 knockdown promotes a robust senescence phenotype in ovarian cancer cells, characterized by multiple hallmarks of this cellular state.

### YTHDC1 regulates telomere length and cellular senescence by modulating TERT expression

**Fig. 4:**
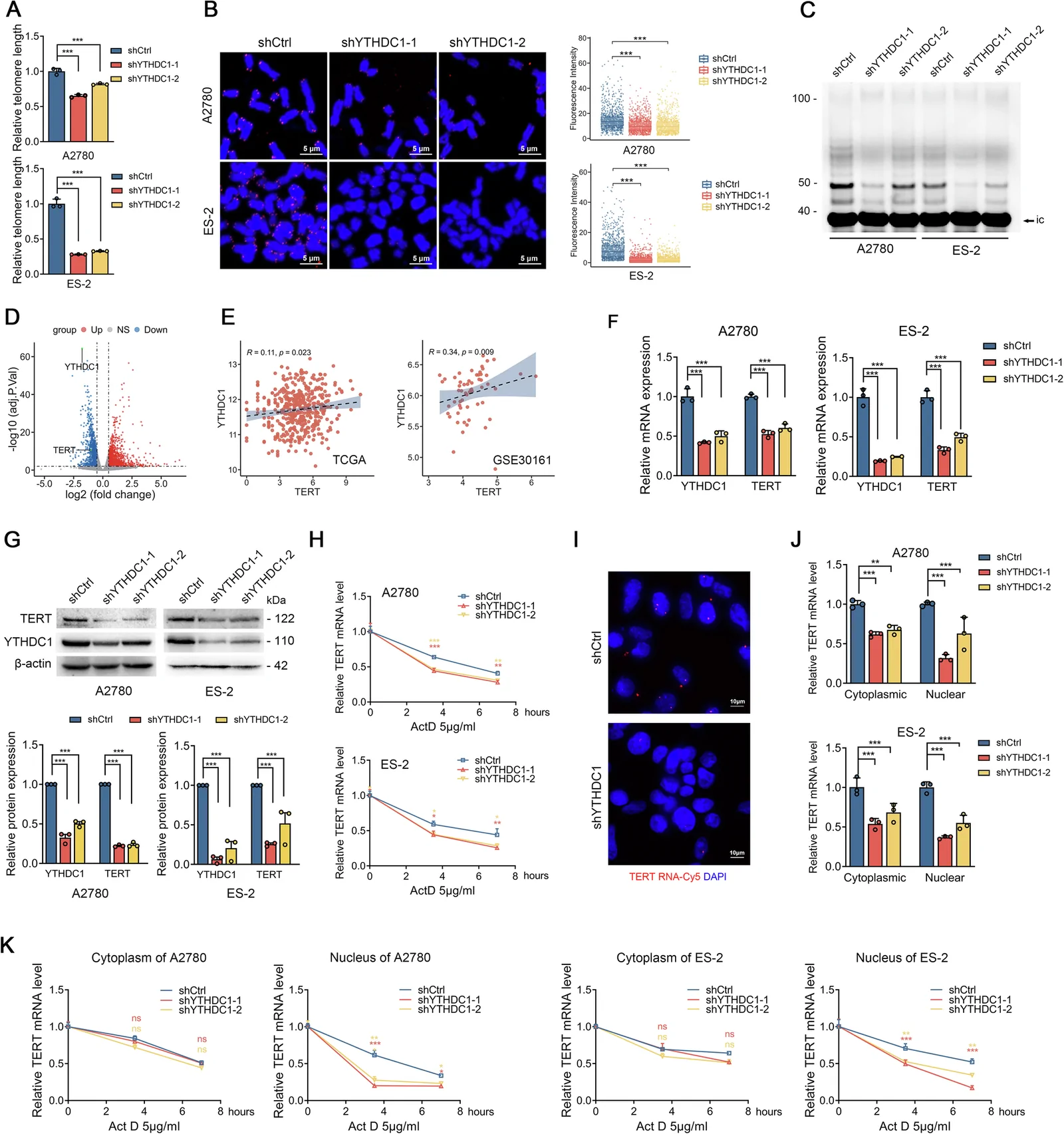
YTHDC1 regulates telomere length and cellular senescence by stabilizing TERT mRNA. **A** Relative telomere length (T/S ratio) measured by qPCR in A2780 and ES-2 cells, *n* = 3. **B** q-FISH analysis of metaphase spreading telomeres in A2780/ES-2 cells with YTHDC1 knockdown. Representative images (left) and quantification (right) of Telc-Cy3 fluorescence intensity. Telc-Cy3 (red), chromosomes (DAPI, blue). **C** Telomerase activity detected by TRAP assay; ic, internal control. **D** Volcano plot of differentially expressed genes from RNA-seq showing YTHDC1 knockdown effect with TERT highlighted among downregulated genes. **E** Correlation analysis demonstrating that YTHDC1 expression is positively correlated with TERT mRNA expression in TCGA and GEO datasets. **F** qPCR analysis of TERT mRNA expression in A2780 and ES-2 cells following YTHDC1 knockdown, *n* = 3. **G** Western blot analysis of TERT protein levels in YTHDC1 knockdown cells. **H** TERT mRNA stability assay. Cells were treated with Act D (5 μg/ml) for the indicated times, and TERT transcript levels were quantified by qRT–PCR, *n* = 3. **I** AsmFISH detection of TERT transcripts (red) in ES-2 cells. **J** qPCR quantification of TERT mRNA in cytoplasmic and nuclear fractions from control and YTHDC1-knockdown cells, *n* = 3. **K** TERT mRNA decay curves in cytoplasmic (left) and nuclear (right) fractions of A2780 and ES-2 cells following Act D treatment, *n* = 3. Data are presented as mean ± SD, Correlations were assessed by Spearman correlation. For decay/time-course analyses, two-way ANOVA with Dunnett’s test was used; for endpoint comparisons, two-sided unpaired Student’s t-test (two groups) or one-way ANOVA with Tukey’s test ( ≥ 3 groups) was used. ns, not significant. *P < 0.05; **P < 0.01; ***P < 0.001.

Telomere shortening is the hallmark of cellular senescence [17]. In our study, YTHDC1 knockdown led to pronounced telomere shortening (Fig. 4A, B) and impaired telomerase activity (Fig. 4C). Telomerase consists of the telomerase RNA component (TERC) and the catalytic subunit TERT, and reactivation of TERT represents a principal mechanism for telomerase hyperactivation in malignant cells [18]. Transcriptomic profiling revealed TERT as one of the significantly downregulated genes upon YTHDC1 silencing (Fig. 4D). Consistently, analysis of TCGA and GEO datasets demonstrated a positive correlation between YTHDC1 and TERT expression in ovarian tumors (Fig. 4E). Moreover, both TERT mRNA and protein levels were markedly reduced following YTHDC1 knockdown (Figs. 4F, G and S2) across four ovarian cancer cell lines (A2780, ES-2, OVCAR3, and SKOV3). Collectively, these findings establish YTHDC1 as a critical post-transcriptional regulator of TERT expression.

We next explored how YTHDC1 regulates TERT. Given the established roles of YTHDC1 in RNA metabolism [19, 20], we first examined potential effects on transcriptional and splicing regulation. Luciferase reporter assays detected no change in TERT promoter activity upon YTHDC1 depletion (Fig. S3A). Furthermore, YTHDC1 knockdown did not alter the splicing ratio of functional full-length TERT (TERT-FL) to non-functional TERT-β [21] (Fig. S3B), indicating that YTHDC1 does not regulate TERT at the transcriptional or splicing level. We then assessed TERT mRNA stability using actinomycin D (ActD) assays. YTHDC1 knockdown accelerated TERT mRNA decay in both A2780 and ES-2 cells (Fig. 4H), indicating a role for YTHDC1 in maintaining mRNA stability. To define the subcellular basis of this effect, we examined TERT RNA distribution and compartment-specific mRNA stability. AsmFISH revealed a global reduction in TERT RNA signals in both the nucleus and cytoplasm upon YTHDC1 knockdown (Fig. 4I), which was confirmed by fractionation-qPCR (Fig. 4J; fractionation controls in Fig. S3C). Notably, compartment-specific mRNA stability assays demonstrated that YTHDC1 deficiency selectively accelerated the degradation of nuclear TERT mRNA, whereas the decay rate of the cytoplasmic pool remained largely unaffected (Fig. 4K). These findings indicate that the reduction in steady-state TERT levels in both compartments is driven by a primary nuclear stability defect. As a nuclear m6A reader, YTHDC1 directly stabilizes TERT transcripts within the nuclear compartment. Its depletion triggers rapid nuclear mRNA degradation, thereby diminishing the pool of transcripts available for nuclear export. Consequently, cytoplasmic TERT abundance declines as a secondary consequence of reduced nuclear supply rather than altered cytoplasmic turnover. Collectively, our results establish YTHDC1 as a critical post-transcriptional regulator that maintains telomerase activity and prevents telomere-shortening–associated senescence by stabilizing nuclear TERT mRNA.

**Fig. 5:**
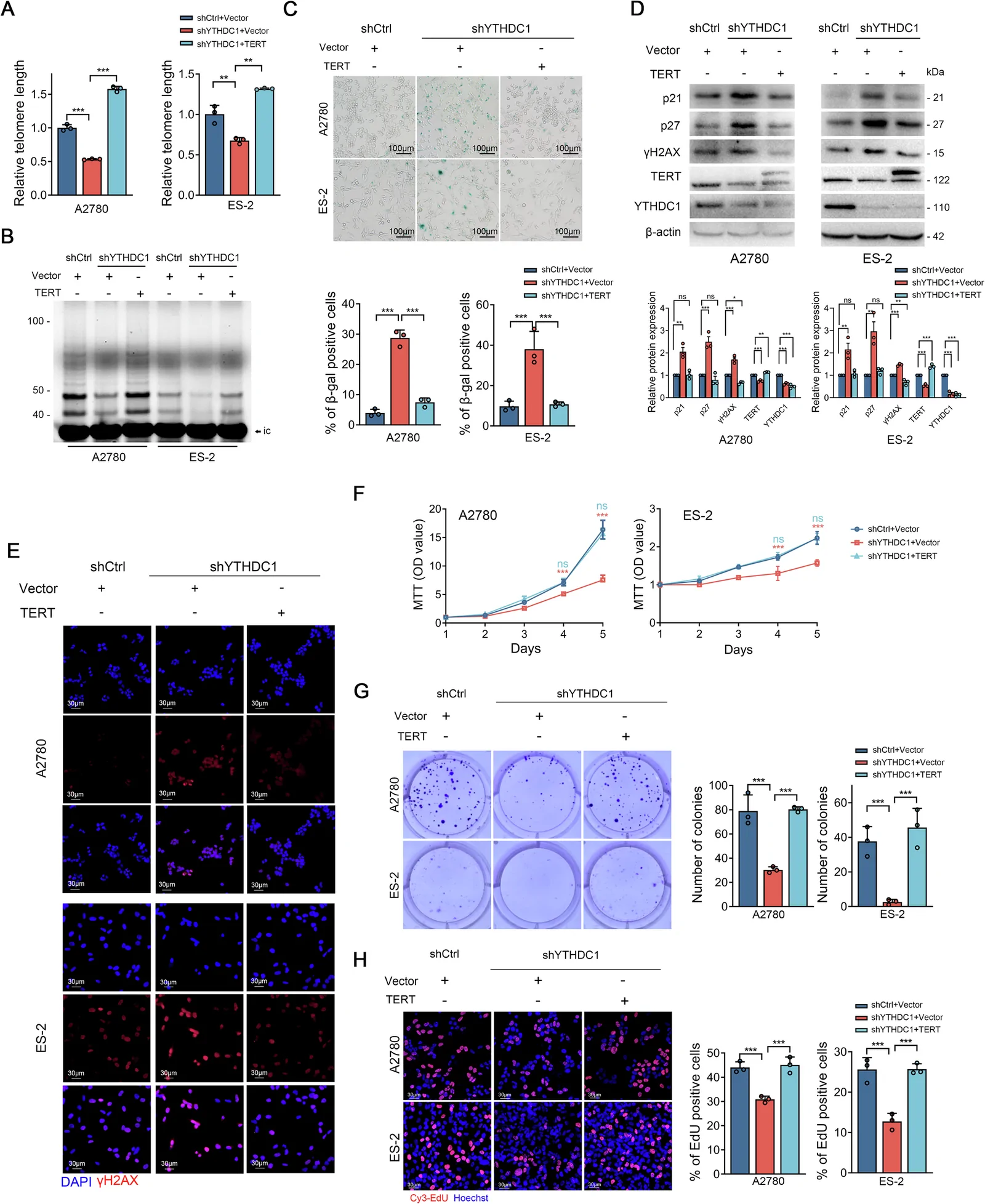
Ectopic TERT expression rescues telomere shortening and senescence induced by YTHDC1 depletion. **A** Relative telomere length (T/S ratio) measured by qPCR, *n* = 3. **B** Telomerase activity determined by TRAP assay. **C** Representative images of β-galactosidase staining and quantitative analysis, *n* = 3. **D** Western blot analysis of TERT and cell senescence-related proteins (p21, p27, γH2AX) in A2780 and ES-2 cells: Representative immunoblots (top) and densitometric quantification (bottom), *n* = 3. **E** Immunofluorescence staining to detect γH2AX (red) expression in A2780 and ES-2 cells; nuclei were counterstained with DAPI. **F** Cell proliferation assessed by MTT assays in A2780 and ES-2 cells, *n* = 5. **G** Colony formation assay of YTHDC1 knockdown cells with or without TERT reconstitution and corresponding quantitative analysis, *n* = 3. **H** Representative images and quantitation of EdU incorporation assay, *n* = 3. Data are presented as the mean ± SD. For MTT curves, two-way ANOVA with Dunnett’s test was used; for other panels, one-way ANOVA with Tukey’s test was used. ns, not significant. * *P* < 0.05; ** *P* < 0.01; *** *P* < 0.001.

To establish TERT as the functional downstream effector, we performed rescue experiments. TERT overexpression in YTHDC1-knockdown cells restored telomere length and telomerase activity (Fig. 5A, B), suppressed senescence (Fig. 5C–E), and rescued proliferation defects (Fig. 5F–H). These rescue experiments collectively demonstrate that TERT is a crucial downstream mediator of YTHDC1 functions in maintaining telomere length, preventing cellular senescence, and promoting cancer cell growth.

### YTHDC1 regulates TERT expression and cellular senescence through its m6A-binding function

**Fig. 6:**
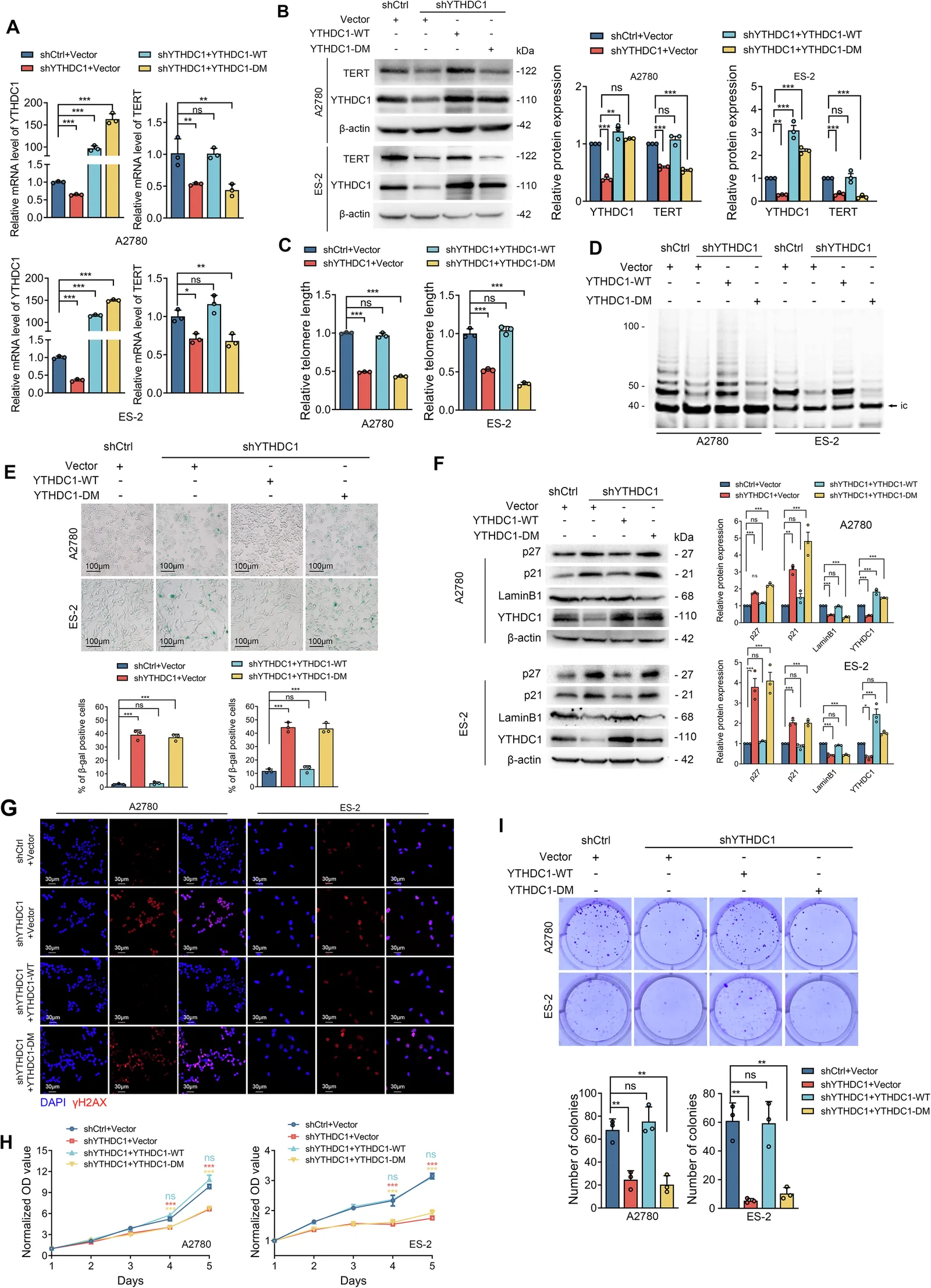
YTHDC1 regulates TERT expression and cellular senescence through its m6A-binding function. **A** qPCR analysis of YTHDC1 and TERT mRNA levels in A2780 and ES-2 cells, *n* = 3. **B** Western blot analysis showing YTHDC1 and TERT protein levels in A2780 and ES-2 cells under the indicated conditions: Representative immunoblots (left) and densitometric quantification (right), *n* = 3. **C** Relative telomere length (T/S ratio) measured by quantitative PCR, *n* = 3. **D** Telomerase activity assessed by TRAP assay. **E** Representative images of β-galactosidase staining and quantitative analysis. **F** Western blot analysis of senescence-associated markers in the indicated conditions. **G** Representative immunofluorescence images of γH2AX (red) expression in the indicated cell lines. Nuclei were counterstained with DAPI (blue). **H** Cell proliferation assessed by MTT assays, *n* = 5. **I** Colony formation assay to assess cell proliferation and corresponding quantitative analysis, *n* = 3. Data are expressed as means ± SD. For MTT curves, two-way ANOVA with Dunnett’s test was used; for other panels, one-way ANOVA with Tukey’s test was used. ns, not significant. * *P* < 0.05; ** *P* < 0.01; *** *P* < 0.001.

To determine whether YTHDC1 regulates TERT expression and cellular senescence in an m6A-dependent manner, we reintroduced wild-type YTHDC1 (YTHDC1-WT) or an m6A-binding-deficient mutant (YTHDC1-DM) [22] into YTHDC1-knockdown cells. YTHDC1-WT, but not YTHDC1-DM, restored TERT mRNA and protein expression (Fig. 6A, B), indicating that m6A recognition is essential for YTHDC1-mediated TERT regulation. Consistently, YTHDC1-WT, but not the mutant, rescued telomere length and telomerase activity (Fig. 6C, D), and reversed senescence phenotypes, as shown by reduced β-galactosidase staining, decreased p21, p27 and γH2AX, and restored Lamin B1 levels (Fig. 6E–G). Functionally, only YTHDC1-WT restored cell proliferation in growth and colony formation assays (Fig. 6H, I). These results collectively demonstrate that YTHDC1’s m6A-binding function is essential for maintaining TERT expression, regulating telomerase activity, preventing cellular senescence, and ultimately promoting cell proliferation in ovarian cancer cells.

### YTHDC1 binds to m6A-modified TERT mRNA to regulate its expression in ovarian cancer

**Fig. 7:**
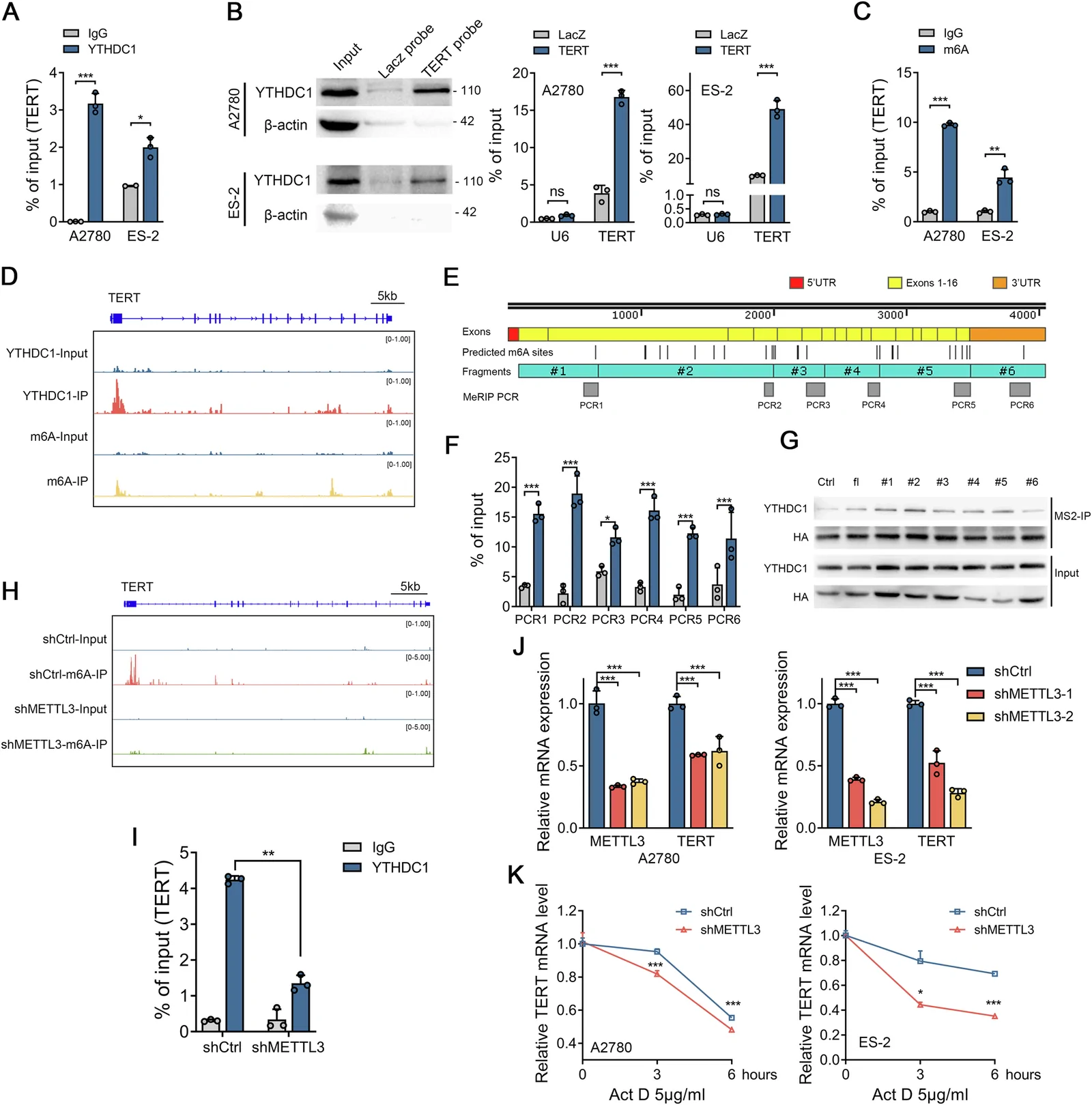
YTHDC1 recognizes m6A-modified TERT mRNA to regulate its stability. **A** RNA immunoprecipitation (RIP)-qPCR analysis showing enrichment of TERT mRNA in A2780 and ES-2 cells using anti-YTHDC1 antibodies compared to IgG controls, *n* = 3. **B** (Left) Representative immunoblots from biotin-labeled RNA pull-down assays showing YTHDC1 protein enrichment with TERT probe versus LacZ control probe in A2780 and ES-2 cells. (Right) Quantification of RNA pull-down assays showing relative enrichment of TERT mRNA versus U6 (negative control), *n* = 3. **C** MeRIP-qPCR analysis of non-fragmented RNA demonstrating m6A enrichment on TERT mRNA in A2780 and ES-2 cells, *n* = 3. **D** Integrative genomic view of RIP-seq (GSE193156) and MeRIP-seq (GSE193155) profiles showing overlapping YTHDC1 binding peaks and m6A modification sites along TERT mRNA. **E** Schematic representation of TERT mRNA structure with 22 predicted m6A modification sites identified by SRAMP algorithm, and six fragments used for MeRIP-qPCR and MS2-TRAP analyses. **F** MeRIP-qPCR analysis of fragmented TERT mRNA in A2780 cells confirming m6A enrichment at predicted sites across six regions (PCR1-PCR6), *n* = 3. **G** MS2-tagged RNA affinity purification (MS2-TRAP) assays identifying YTHDC1 binding regions across six TERT mRNA fragments. **H** MeRIP-seq profiles (GSE110320) showing reduced m6A peaks in METTL3-knockdown (shMETTL3) cells compared to control (shCtrl) cells. **I** RIP-qPCR demonstrating decreased interaction between YTHDC1 and TERT mRNA in METTL3-knockdown A2780 cells compared to control cells. **J** RT-qPCR analysis showing reduced TERT mRNA expression levels in METTL3-knockdown cells in both A2780 and ES-2 cell lines, *n* = 3. **K** TERT mRNA stability assay, *n* = 3. Data are presented as the mean ± SD. For decay/time-course analyses, two-way ANOVA with Dunnett’s test was used; for end-point comparisons, two-sided unpaired Student’s *t* test (two groups) or one-way ANOVA with Tukey’s test ( ≥ 3 groups) was used. ns, not significant. *P < 0.05; **P < 0.01; ***P < 0.001.

To characterize how YTHDC1 interacts with m6A-modified TERT mRNA, we performed RNA immunoprecipitation (RIP) and biotin-labeled RNA pull-down assays, both of which confirmed a direct and specific interaction of YTHDC1 protein and TERT mRNA in A2780 and ES-2 cells (Fig. 7A, B). To characterize the epitranscriptomic landscape of TERT mRNA, we first performed methylated RNA immunoprecipitation (MeRIP)-qPCR on non-fragmented RNA, which revealed significant m6A enrichment in TERT mRNA in both cell lines (Fig. 7C). Integrated analysis of RIP-seq and MeRIP-seq data revealed overlapping peaks of YTHDC1 binding and m6A modifications along the TERT mRNA (Fig. 7D), providing compelling evidence that YTHDC1 binds to m6A-modified sites. To map specific m6A sites, we utilized the SRAMP software, which predicted 22 potential m6A modification sites across TERT mRNA (Fig. 7E and Table S4). MeRIP-qPCR analysis of the fragmented RNA using six primer pairs confirmed significant m6A enrichment across these predicted sites (Fig. 7E, F). To precisely define YTHDC1 binding regions, we performed MS2-tagged RNA affinity purification (MS2-TRAP) assays with six distinct TERT mRNA fragments spanning the full transcript (Fig. 7E). This analysis revealed that YTHDC1 binds to multiple regions of TERT mRNA, with the highest enrichment observed for fragment #2 (exons 2–4), which harbors the highest density of predicted m6A sites (Fig. 7G).

To establish the functional importance of m6A modifications in TERT regulation, we conducted METTL3 knockdown experiments. MeRIP-seq analysis showed significantly reduced m6A enrichment peaks in METTL3-knockdown cells, particularly in exons 1-2 (Fig. 7H), suggesting METTL3 mediated m6A modification in TERT. RIP assays demonstrated a significantly decreased interaction between YTHDC1 and TERT mRNA following METTL3 knockdown (Fig. 7I), confirming that METTL3-mediated m6A deposition is a prerequisite for YTHDC1 recruitment. Moreover, METTL3 knockdown significantly reduced TERT expression (Fig. 7J) and accelerated TERT mRNA degradation (Fig. 7K) in A2780 and ES-2 cell lines. These findings established that YTHDC1 recognizes multiple METTL3-dependent m6A modifications across TERT mRNA to regulate its stability in ovarian cancer cells.

### Senolytic drug ABT-263 potentiates the anti-tumor effects of YTHDC1 depletion by eliminating senescent ovarian cancer cells

These results showed that YTHDC1 knockdown induced cellular senescence in ovarian cancer cells, suggesting a novel therapeutic avenue. However, while senescence halts proliferation, persistent senescent cells can promote tumor progression through SASP and acquired stem-like properties. Consequently, strategies for the selective elimination of these senescent cells are warranted. In this study, we investigated whether the senolytic agent ABT-263 can augment the therapeutic efficacy of YTHDC1 knockdown.

**Fig. 8:**
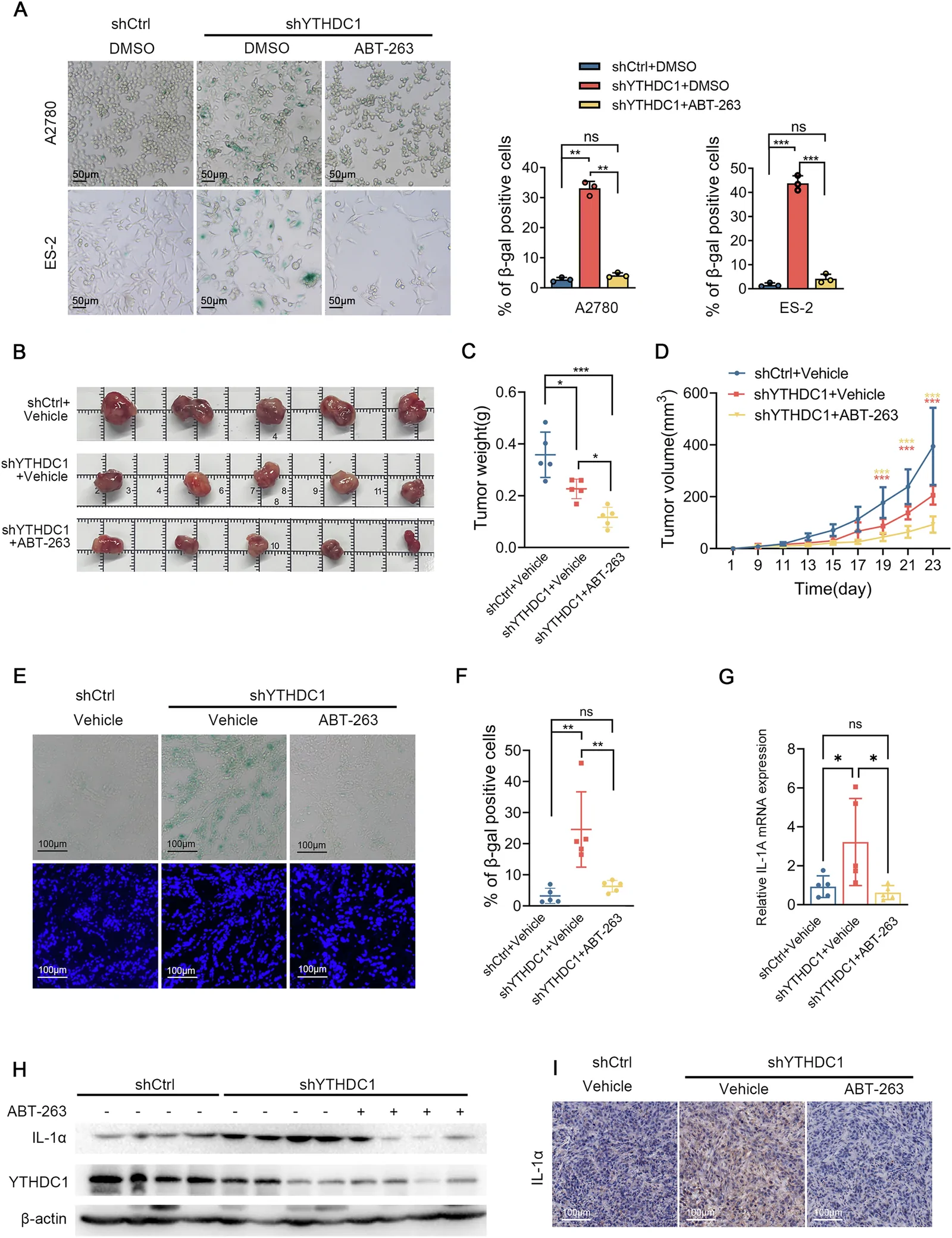
Combined YTHDC1 depletion and ABT-263 treatment synergistically suppress ovarian cancer. **A** Representative images (left) and quantification (right) of SA-β-Gal staining in A2780 and ES-2 cells following YTHDC1 knockdown and ABT-263 treatment, *n* = 3. Images (**B**), tumor weights (**C**), and tumor growth curves (**D**) of ES-2 xenograft tumors, *n* = 5. **E** Representative images of SA-β-gal staining (top; brightfield) and DAPI staining for nuclei (bottom; blue fluorescence) in tissue sections from ES-2 xenograft tumors. **F** Quantification of the percentage of SA-β-gal-positive cells in tumor sections, *n* = 6. **G** Relative mRNA levels of IL1A in ES-2 xenograft tumor, *n* = 6. **H** Western blot analysis of IL-1α protein levels in lysates from ES-2 xenograft tumors. **I** Immunohistochemical staining of IL-1α in tumor sections from xenograft models. Data are presented as the mean ± SD. For tumor growth curves, two-way ANOVA with Dunnett’s test was used; for other panels, one-way ANOVA with Tukey’s test was used. ns, not significant; *P < 0.05; **P < 0.01; ***P < 0.001.

In vitro, ABT-263 reduced the proportion of SA-β-gal–positive senescent cells following YTHDC1 knockdown in A2780 and ES-2 cells (Fig. 8A). For in vivo validation, treatment was initiated once ES-2 xenografts were established (approximately 100 mm³; day 9 post-implantation). Mice then received ABT-263 (50 mg/kg/day) or vehicle via oral gavage for 14 consecutive days, following established preclinical protocols that balance senolytic efficacy and tolerability [23–25]. No evident systemic toxicity was observed based on body-weight monitoring and endpoint histology of liver/kidney (Fig. S4). Concomitant YTHDC1 knockdown and ABT-263 administration markedly suppressed tumor growth, characterized by significantly reduced tumor volumes and weights at the study endpoint relative to YTHDC1 knockdown alone (Fig. 8B–D). Histological analysis confirmed that ABT-263 effectively cleared YTHDC1 depletion–induced senescent cells in vivo (Fig. 8E, F). Moreover, ABT-263 attenuated SASP triggered by YTHDC1 loss, reducing the expression of IL1A mRNA and IL-1α protein (Fig. 8G–I).

These findings demonstrate that senolytic targeting with ABT-263 effectively eliminates YTHDC1 knockdown–induced senescent cells, alleviates SASP, and enhances antitumor efficacy, supporting a promising combinatorial strategy for ovarian cancer treatment.

## Discussion

Ovarian cancer remains one of the most lethal gynecologic malignancies, and its high recurrence rate highlights the urgent need for novel therapeutic strategies. Our study identifies YTHDC1 as a pivotal oncogenic regulator that sustains telomerase activity and prevents cellular senescence. YTHDC1 is markedly upregulated in ovarian cancer and closely associated with poor prognosis and advanced disease stages. Mechanistically, YTHDC1 binds to m6A-modified TERT transcripts, stabilizing their expression and maintaining telomere homeostasis. Loss of YTHDC1 compromises telomerase activity, promotes telomere attrition, and triggers senescence. Notably, the combination of YTHDC1 depletion with the senolytic compound ABT-263 effectively eliminates senescent tumor cells, revealing a potential therapeutic synergy. These findings delineate an epitranscriptomic mechanism that integrates m6A recognition with telomerase regulation, offering new insight into senescence control and cancer intervention.

YTHDC1 orchestrates multiple layers of RNA metabolism through selective recognition of m6A-modified transcripts. Predominantly localized in the nucleus [26], YTHDC1 governs pre-mRNA processing and nuclear export. It modulates alternative splicing by recruiting SRSF3 while antagonizing SRSF10 [26], and promotes mRNA export through an SRSF3–TREX–NXF1 axis [20]. In cancer biology, YTHDC1 has been implicated in various malignancies through these regulatory mechanisms. It enhances SMAD3 mRNA export to promote metastasis in triple-negative breast cancer [27], and alters OIP5/GPD1L expression balance in gastric cancer [28], and cooperates with AURKA to produce oncogenic RBM4-S isoforms in lung cancer [29]. Distinct from these established functions, our study identifies a novel nuclear mRNA-stability mechanism centered on TERT. YTHDC1 depletion does not alter TERT promoter activity or TERT alternative splicing (TERT-FL vs. TERT-β), but instead accelerates TERT mRNA decay, with the dominant defect localized to the nuclear compartment. Although YTHDC1 has been reported to regulate RNA stability in other biological contexts (e.g., Beclin1 stabilization in macrophages and PTEN destabilization in neurons [30], its contribution to cancer-relevant mRNA stability has been less defined. Our findings reveal TERT as the first oncogenic target of YTHDC1-mediated stabilization, linking epitranscriptomic control to telomerase maintenance and senescence suppression. This mechanism expands the functional spectrum of YTHDC1 beyond splicing and export, positioning it as a central regulator that couples m6A recognition with telomere homeostasis to sustain cancer cell proliferation and immortality.

This nuclear TERT-stabilization axis also broadens the current view of how m6A signaling intersects with cellular senescence. The m6A epitranscriptomic pathway has emerged as a central but context-dependent modulator of senescence. In physiological aging, the integrity of the m6A writer complex is essential for delaying senescence, as Lamin A safeguards METTL14 stability to preserve nuclear m6A homeostasis [31]. In certain cancer contexts, such as alternative lengthening of telomeres (ALT) cells, the m6A pathway indirectly suppresses senescence by ensuring telomere stability through YTHDC1-mediated stabilization of TERRA RNA [32]. YTHDC1 may also delay senescence through m6A-independent mechanisms, such as ATR pathway activation in pulmonary fibrosis [33]. Distinct from these, our study uncovers TERT mRNA stability as a direct m6A-dependent effector of YTHDC1 in ovarian cancer, linking epitranscriptomic control to telomerase-dependent senescence evasion.

A key conceptual advance of this study is the compartmentalized nature of m6A reader control over TERT. Our fractionation-based stability assays show that YTHDC1 loss selectively accelerates nuclear TERT mRNA decay, whereas the decay kinetics of the cytoplasmic pool remain largely unchanged (Fig. 4K). This supports a nuclear checkpoint model in which YTHDC1 acts as a critical gatekeeper prior to export. By stabilizing transcripts within the nuclear compartment, YTHDC1 dictates the pool of export-competent TERT mRNA available for translocation, thereby secondarily determining steady-state cytoplasmic TERT abundance (Fig. 4J) without directly altering cytoplasmic turnover. This nuclear positioning distinguishes YTHDC1 from major cytoplasmic m6A reader families (e.g., YTHDF and IGF2BP), which are generally implicated in post-export regulation of translation and/or cytoplasmic mRNA turnover [34–37]. While YTHDC1 acts as a critical nuclear stabilizer of TERT mRNA, we also investigated whether cytoplasmic m6A readers provide an additional layer of post-transcriptional control. Given the diverse roles of cytoplasmic readers in mRNA turnover and translation, it was possible that they could further modulate TERT fate following its export from the nucleus. However, siRNA-mediated silencing of YTHDF1, YTHDF2, or YTHDF3 in A2780 and ES-2 cells failed to significantly alter TERT mRNA or protein expression (Fig. S5). These findings indicate that, at least under basal conditions in these ovarian cancer models, the YTHDF family is dispensable for maintaining TERT abundance. Nevertheless, it is important to note that our findings do not categorically exclude cytoplasmic regulation. While YTHDF-mediated control appears minimal in our systems, other specialized cytoplasmic readers—or even the YTHDFs themselves—might influence TERT mRNA fate under specific physiological contexts, such as cellular stress, altered export rates, or translational rewiring. Whether these cytoplasmic pathways serve as secondary layers of TERT regulation remains an intriguing subject for future investigation, as they would operate through mechanisms distinct from the primary nuclear stabilization step identified here. Furthermore, although YTHDC1 is the most well-characterized nuclear m6A reader, we recognize that the landscape of nuclear m6A-binding proteins is still expanding; whether other, as-yet-unidentified nuclear readers also participate in TERT regulation remains an open and intriguing question for future investigation. Collectively, our results establish YTHDC1 as a unique and critical regulator that governs the “source” of TERT transcripts, a function that appears more dominant than potential cytoplasmic regulation in this setting.

Telomere dysfunction and progressive telomere shortening normally trigger checkpoint activation and permanent arrest, forming an intrinsic tumor-suppressive barrier[38]. However, therapeutic exploitation of telomere dysfunction remains challenging. Immunotherapeutic approaches, such as TERT peptide vaccines, have yielded only marginal clinical efficacy in preliminary trials. Molecular interventions, including small-molecule telomerase inhibitors such as BIBR1532, are constrained by complex pharmacokinetic profiles and potential cytotoxicity [39]. Nucleoside analogs, such as 6-thio-2’-deoxyguanosine, can induce telomere dysfunction, but tumor cells may develop adaptive resistance mechanisms that compromise therapeutic efficacy [40]. Emerging strategies have focused on targeting TERT promoter mutations through innovative techniques, such as CRISPR-mediated correction and MAPK pathway modulation [41], while exploring alternative approaches, such as manipulating TERT splicing factors to generate non-functional transcripts [21]. Our research demonstrated that YTHDC1 knockdown in ovarian cancer cells significantly modulated TERT expression by affecting mRNA stability, consequently reducing cancer cell proliferation both in vitro and in vivo. Although recent studies have identified small-molecule inhibitors of YTHDC1, these compounds currently exhibit suboptimal potency and selectivity, necessitating further development to enhance their therapeutic potential for telomere- and telomerase-related cancer treatments.

While senescence induction provides a barrier to tumor growth, the persistence of senescent cells may paradoxically promote malignancy through SASP, which can enhance stemness [42], drug resistance, tumor invasion [43], and immune evasion [44]. Although YTHDC1 knockdown effectively inhibited the proliferation of ovarian cancer cells, potentially offering an extended therapeutic window for surgical intervention or chemotherapy, the potential adverse effects of senescent cells warrant careful consideration. Senolytic drugs, which selectively eliminate senescent cells, represent a promising adjuvant strategy for enhancing the efficacy of conventional and targeted cancer therapies [45, 46]. In this study, we investigated the therapeutic potential of combining a YTHDC1 knockdown with the senolytic drug ABT-263 to eliminate senescent cancer cells. Our findings demonstrated that ABT-263 effectively eliminated YTHDC1 knockdown-induced senescence in ovarian cancer cells both in vitro and in vivo. This strategy mitigates the risks associated with senescent cells and the secretion of pro-inflammatory factors that promote tumor risk [47, 48] while augmenting the anti-tumor effects of YTHDC1 depletion. However, the clinical translation of senolytics requires further investigation and optimization to ensure efficacy and safety. Nonetheless, this combination approach has strong translational potential and can overcome the limitations of the current ovarian cancer treatments.

In conclusion, this study elucidates YTHDC1’s critical role in stabilizing TERT mRNA and maintaining telomerase activity in ovarian cancer. By demonstrating that YTHDC1 knockdown induces cellular senescence and inhibits the proliferation of cancer cells, this study reveals a novel molecular mechanism that is potentially amenable to therapeutic intervention. A synergistic approach combining YTHDC1 depletion with the senolytic drug ABT-263 effectively suppressed tumor growth in both in vitro and in vivo models. These findings not only reveal a previously uncharacterized function of YTHDC1 and m6A modification but also highlight its potential as a promising therapeutic target in ovarian cancer. This work represents a significant advancement in our understanding of telomere biology and cancer progression and offers new insights into potential treatment strategies.

## Supplementary information

Supplementary Figures and Tables

Supplementary Material and Methods

Original Western Blots

## Data Availability

RNA-seq data have been deposited in NCBI’s Gene Expression Omnibus and are accessible under GEO Series accession number GSE306807. The data reported, and any additional information required to re-analyze the data reported in this study, are available from the corresponding author upon request. Source data were provided in this study.
