# Supplementary material for: YTHDC1 drives senescence evasion in ovarian cancer through m6A-mediated TERT stabilization
Source: Cell Death Dis. 2026 May 30;17(1):953. doi: 10.1038/s41419-026-08896-7 (PMC13748622; doi:10.1038/s41419-026-08896-7)

Source data for Figure 3E

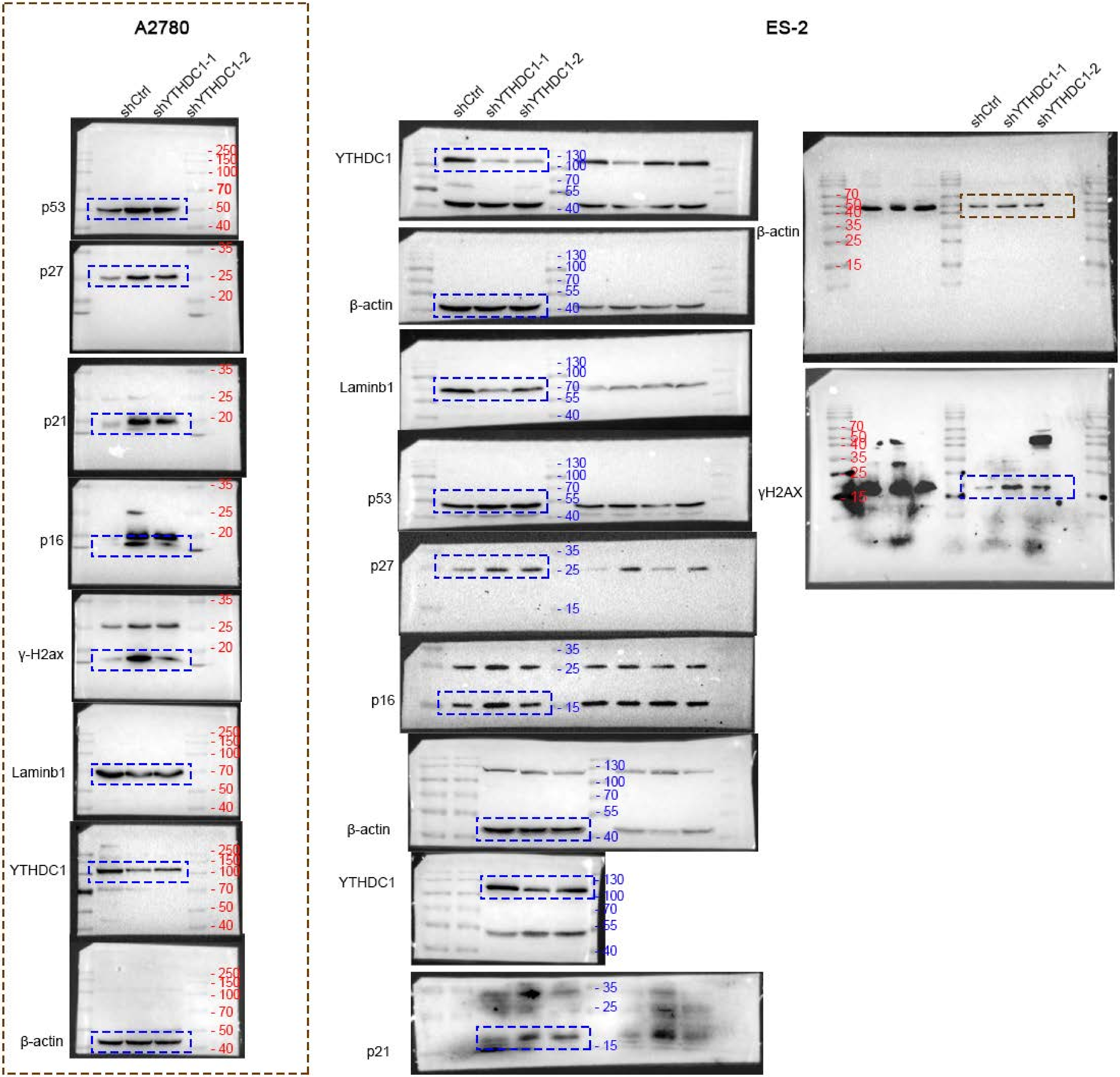

Source data for Figure 4G

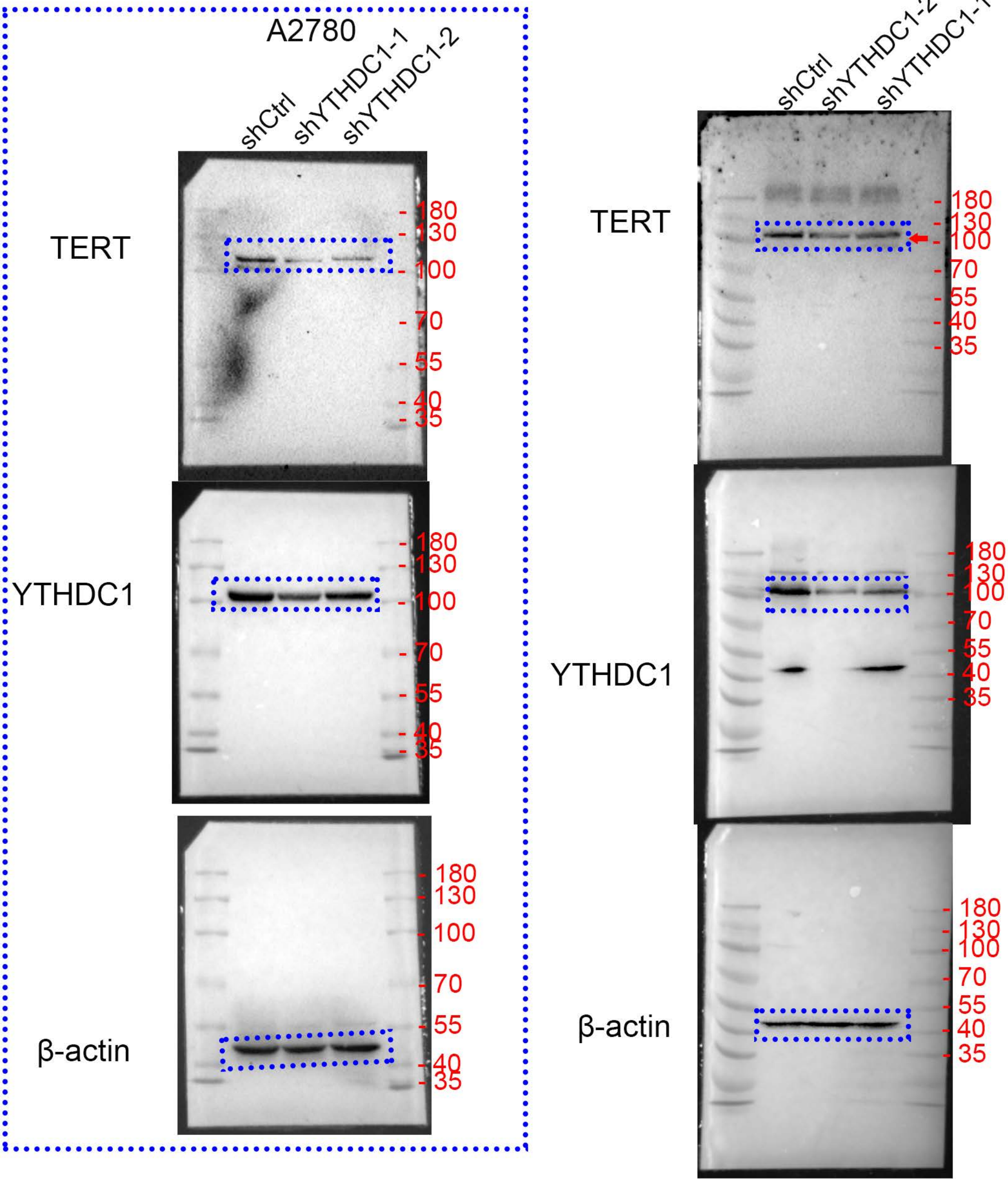

Source data for Figure 5D

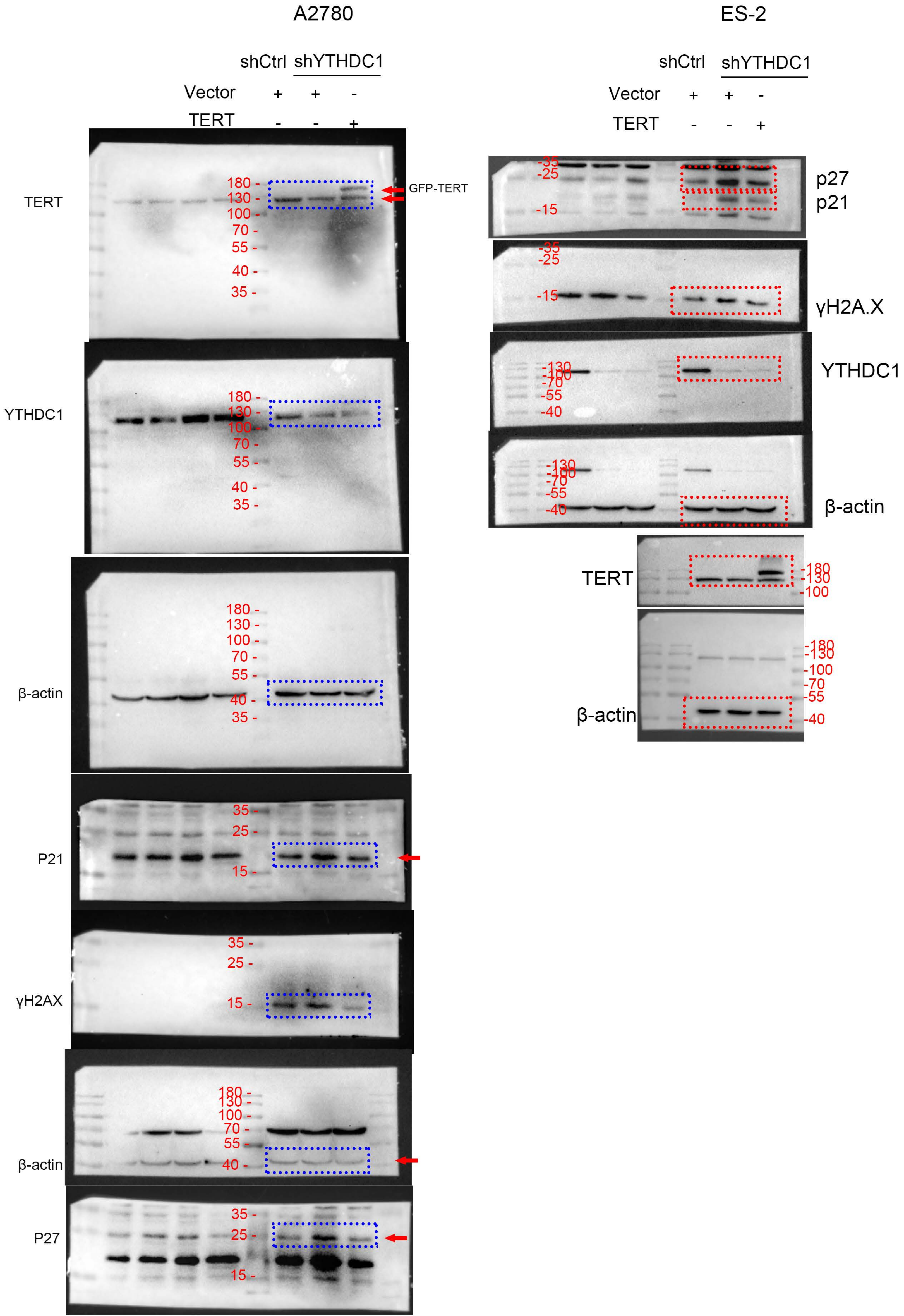

Source data for Figure 6B

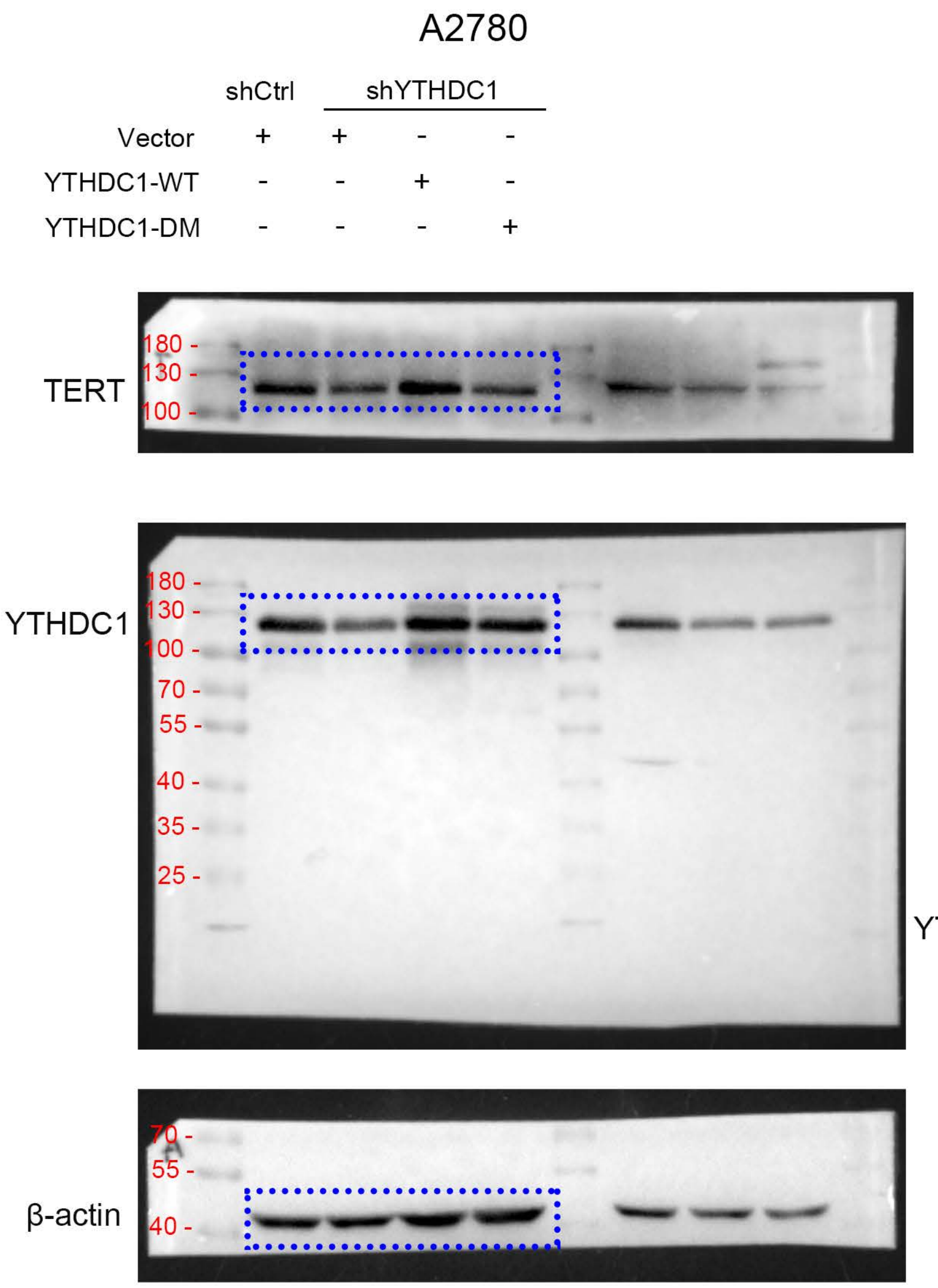

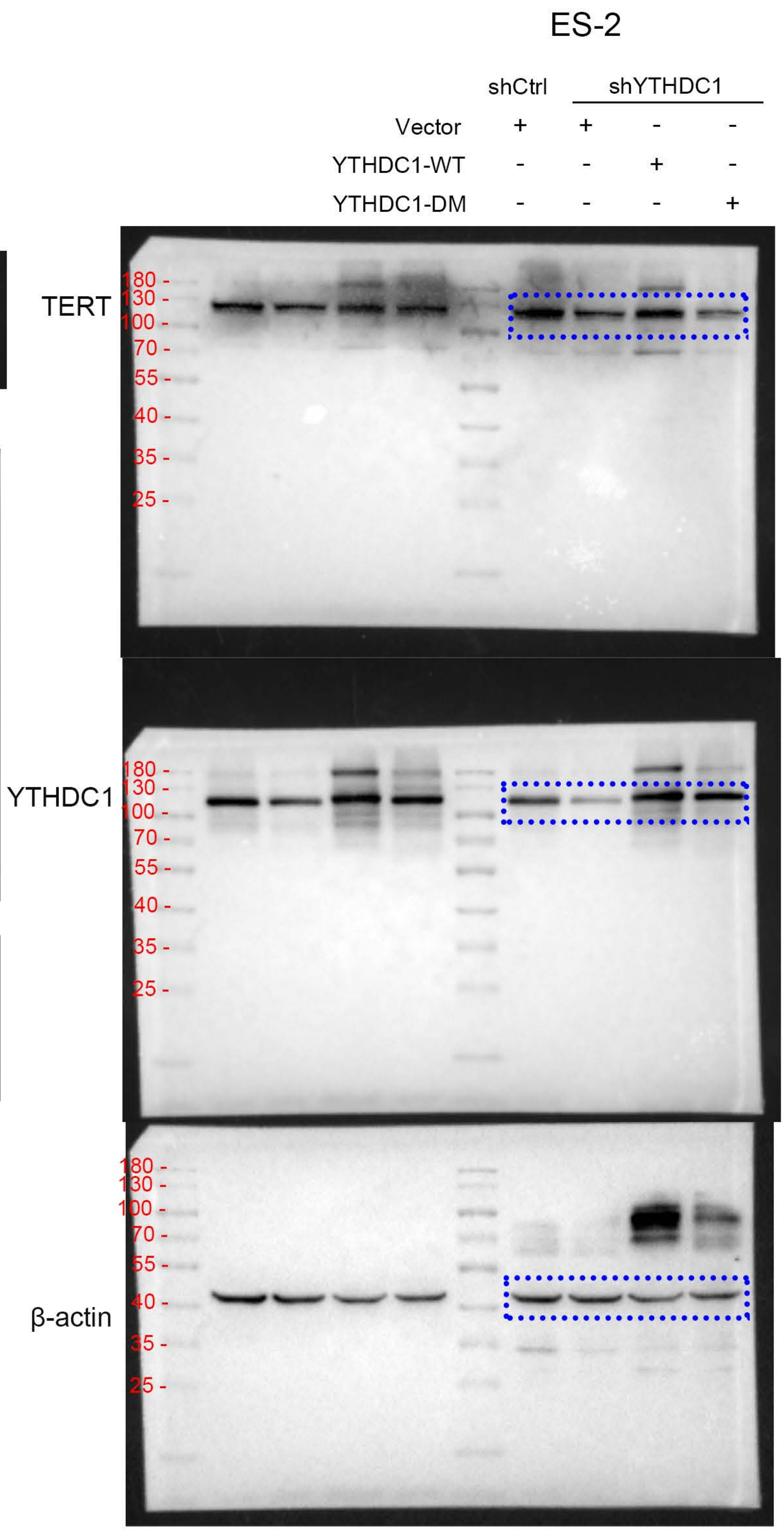

Source data for Figure 6F

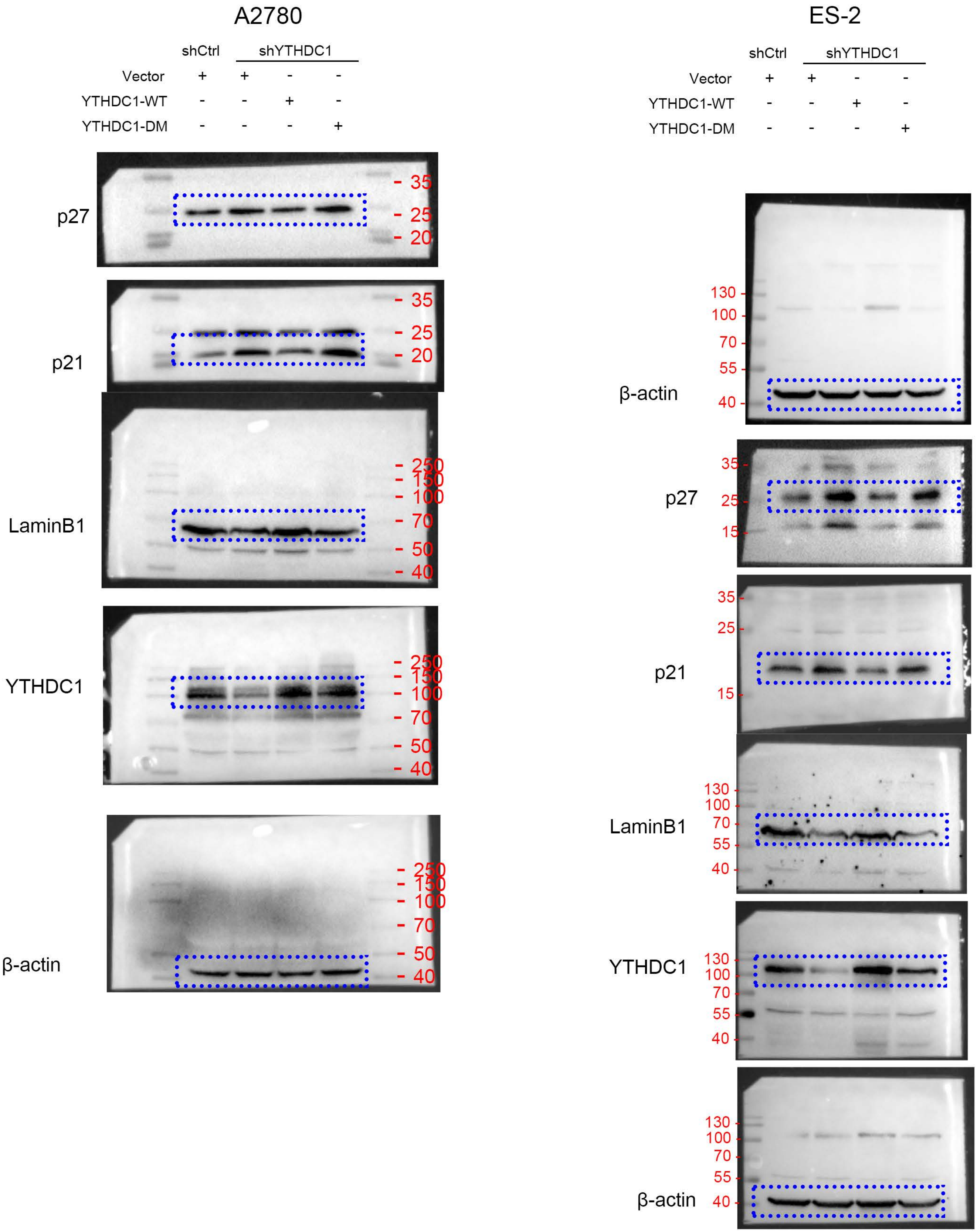

Source data for Figure 7B

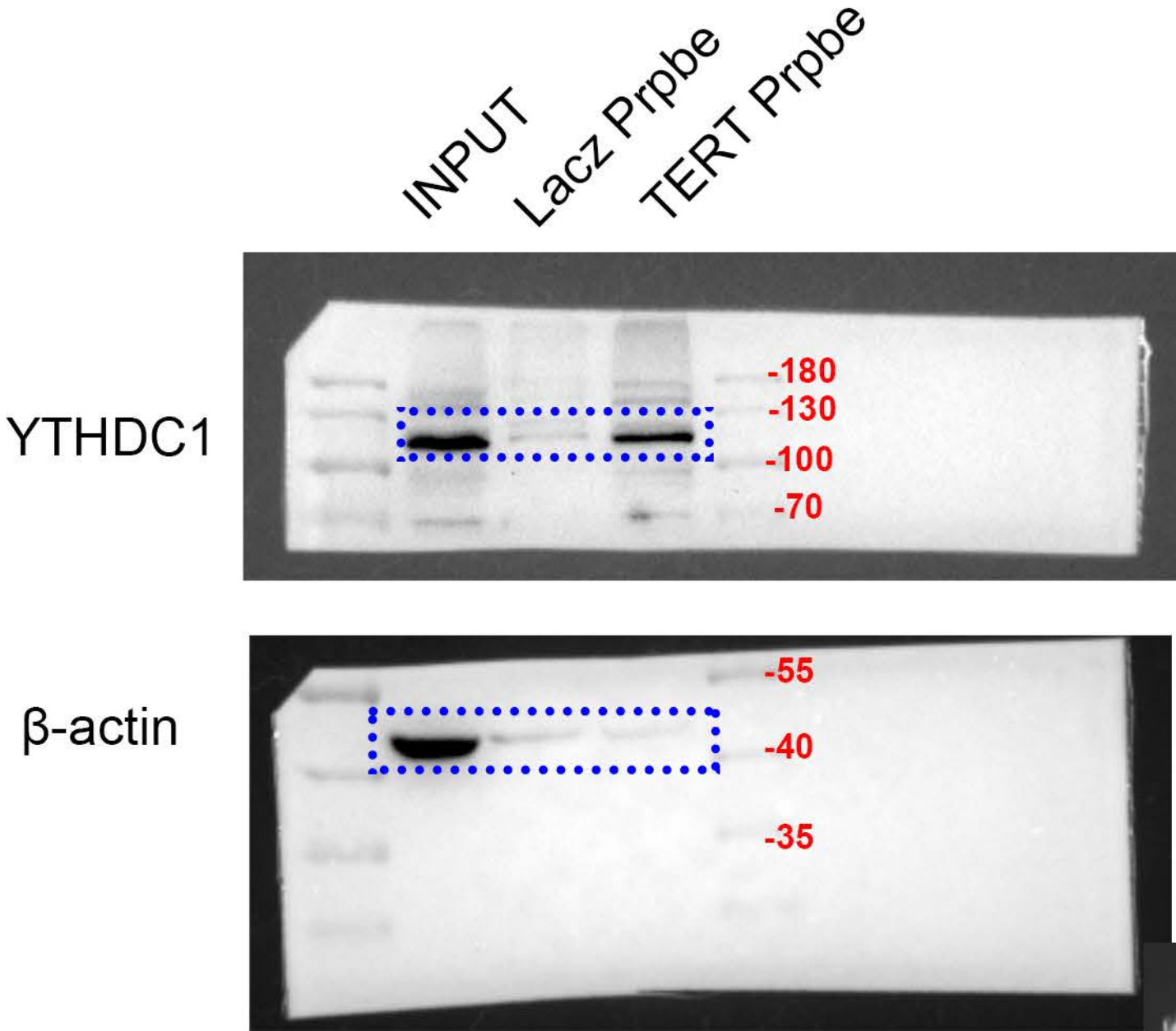

A2780

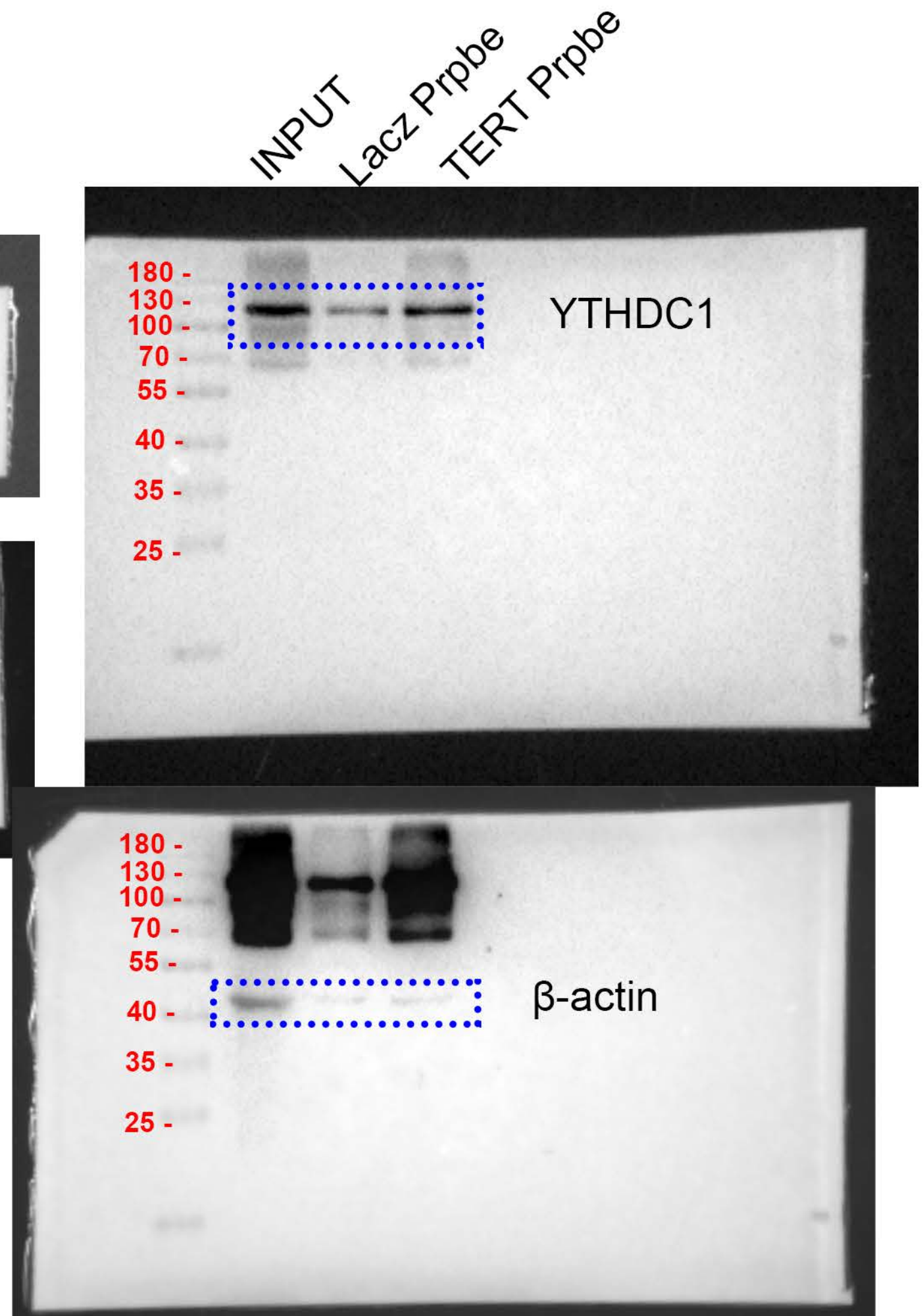

ES-2

Source data for Figure 7G

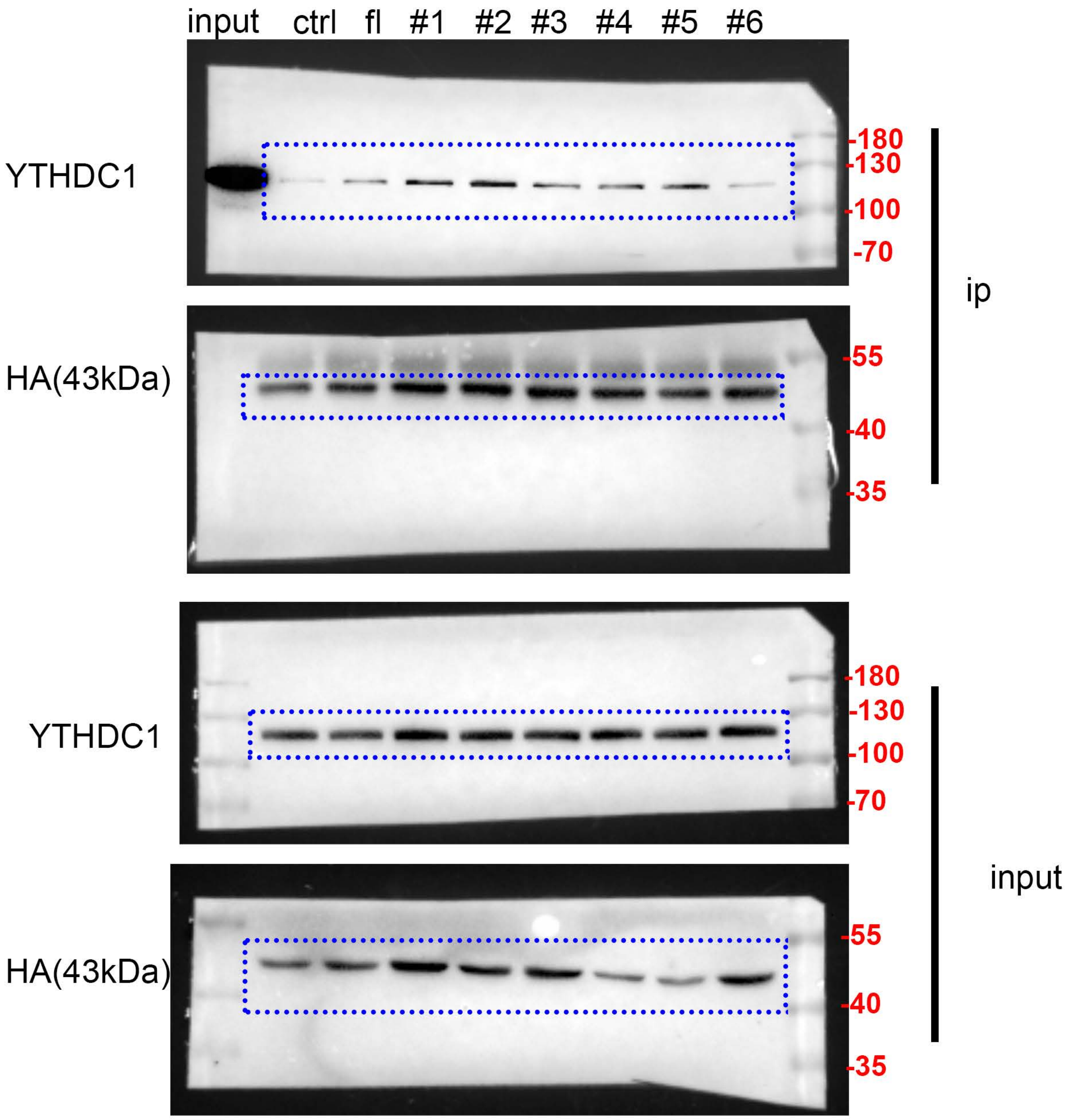

Source data for Figure 8H

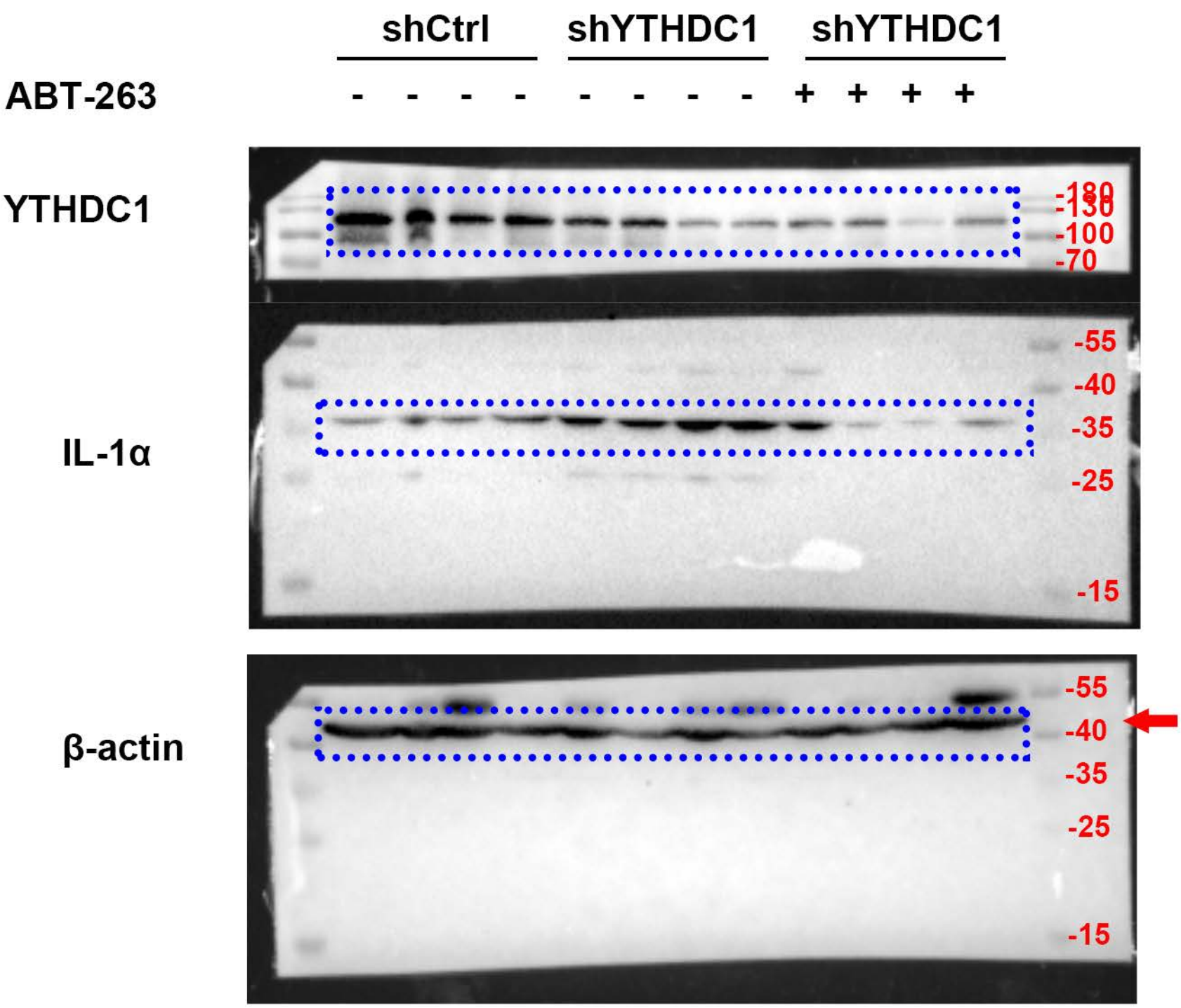

Source data for Figure S1A

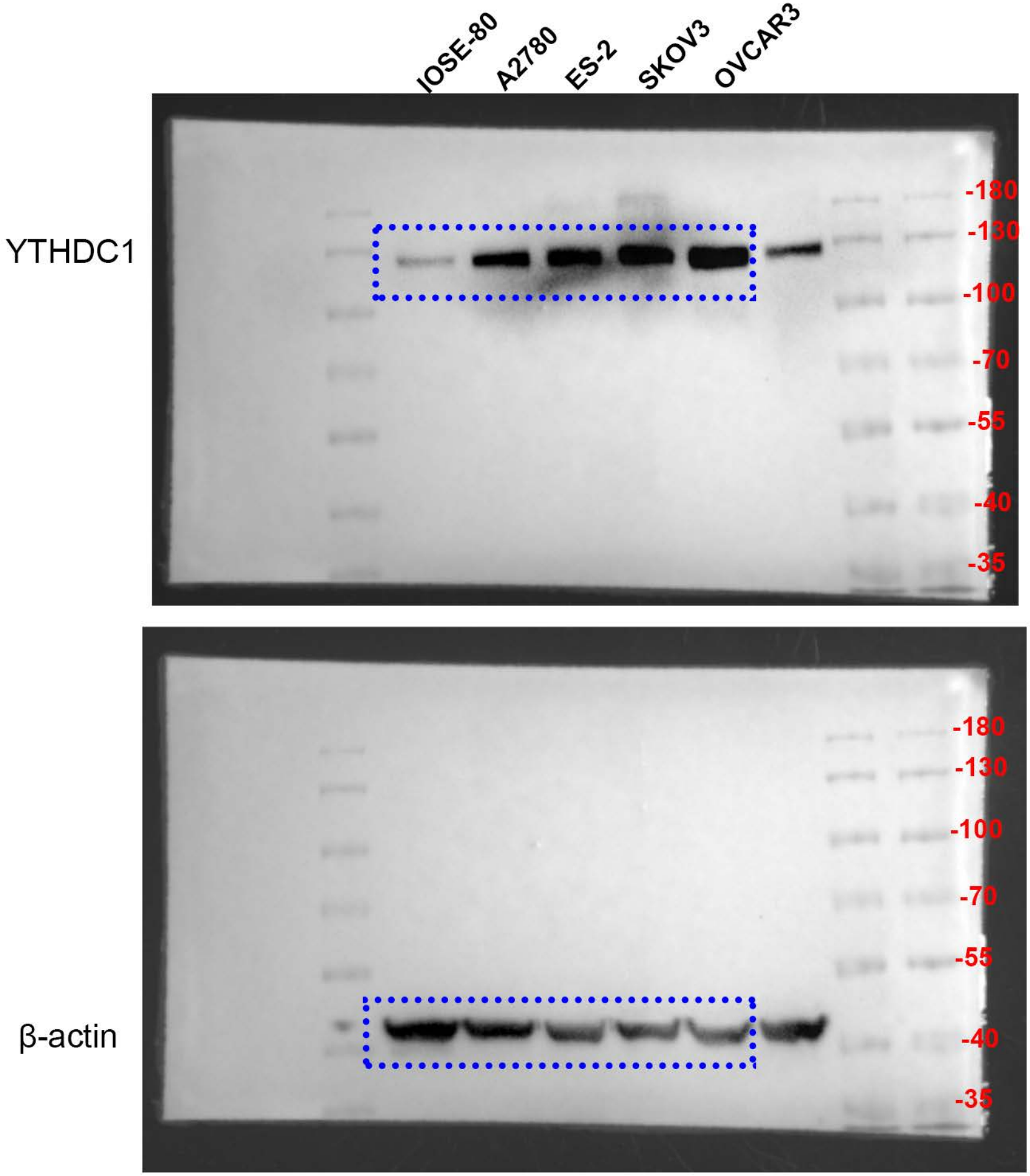

Source data for Figure S1B

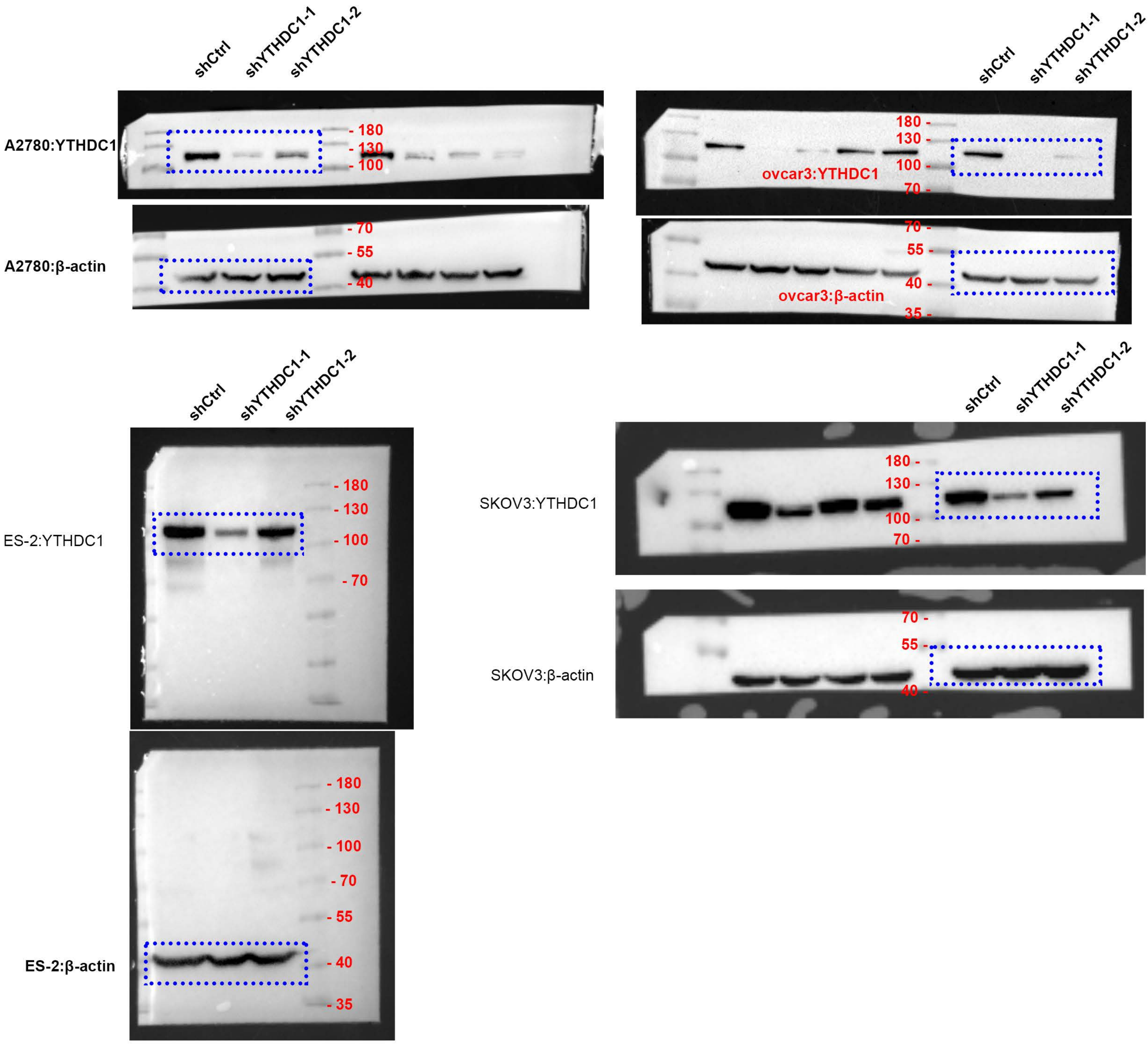

Source data of Figure S2B

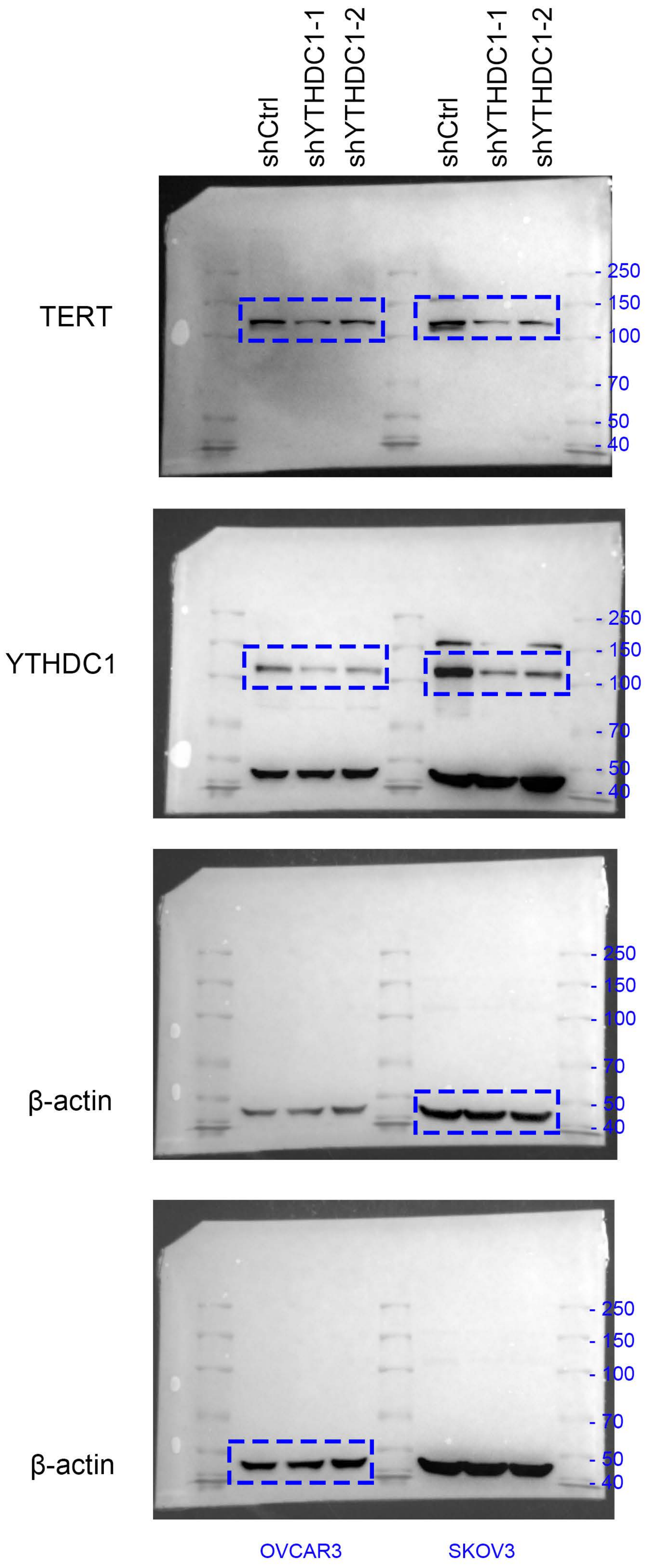

Source data of Figure S5B

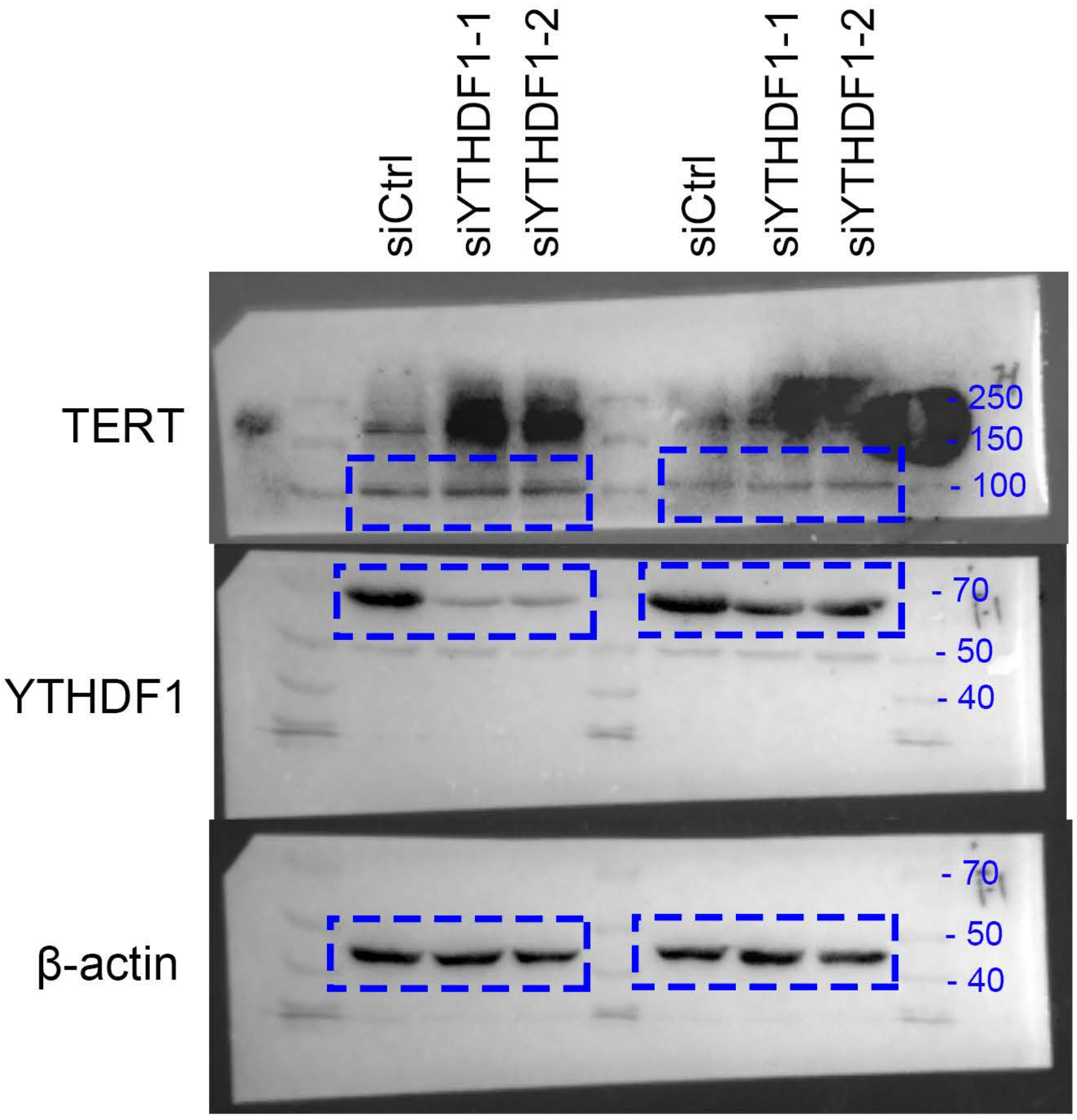

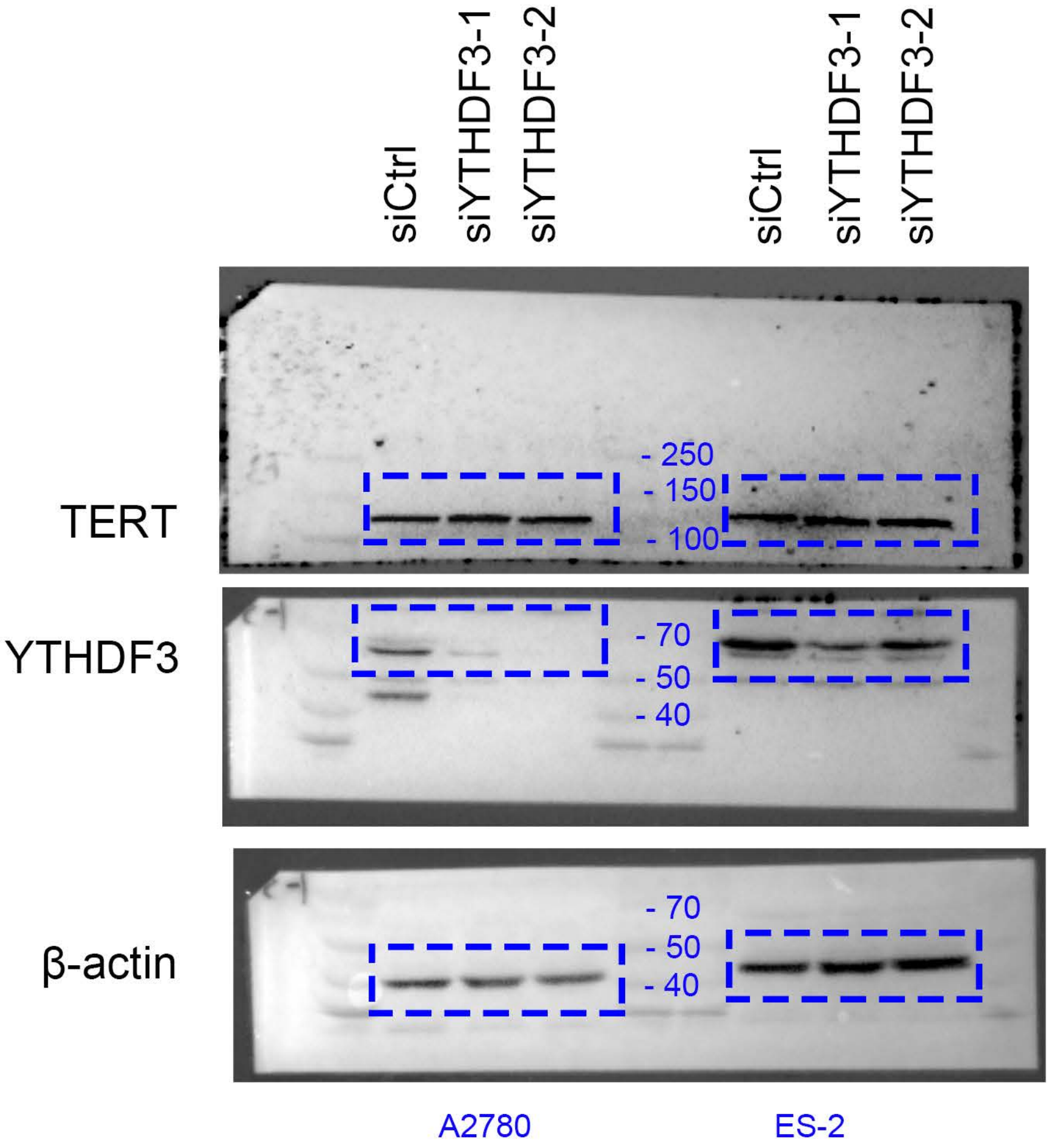

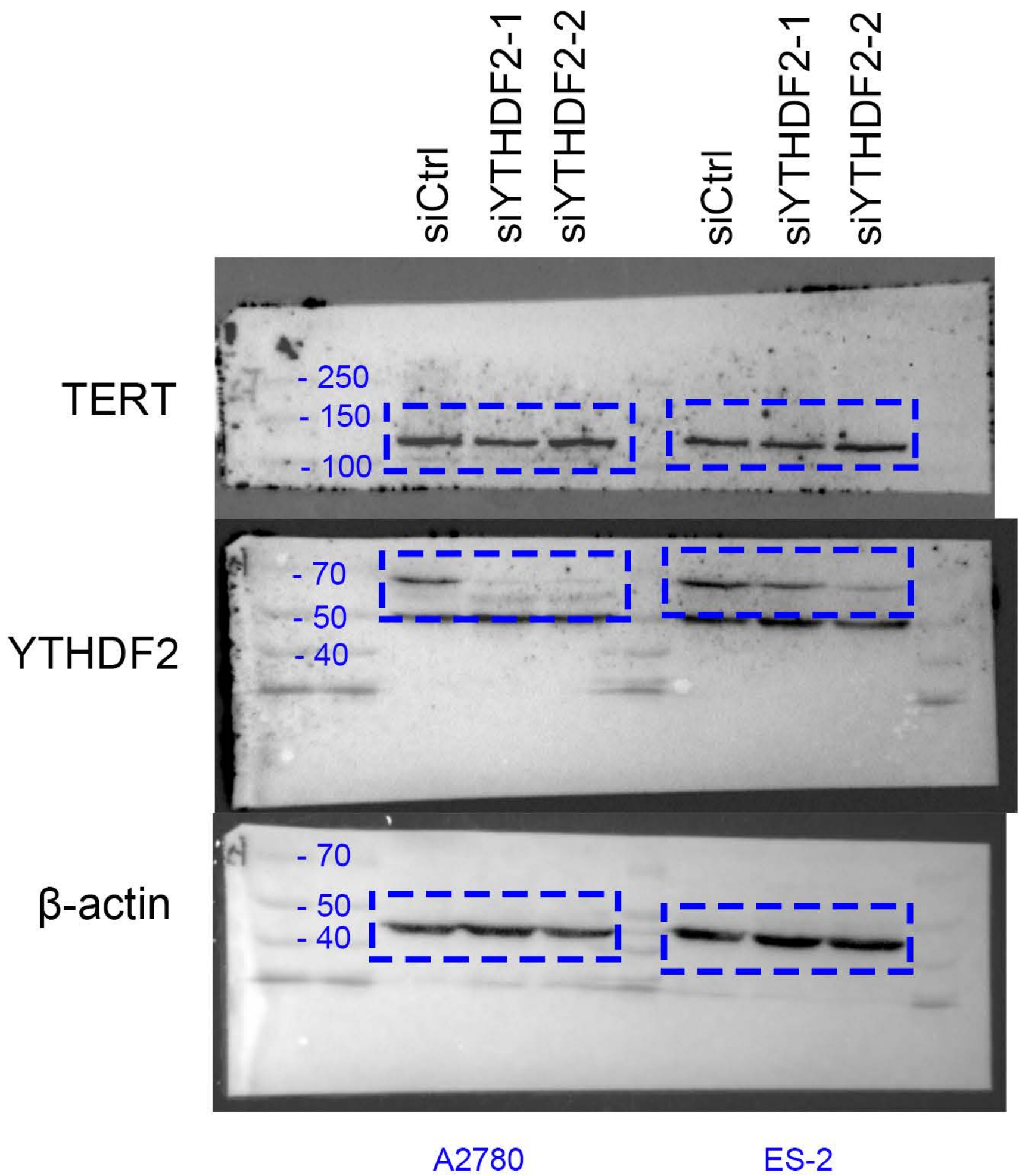

Supplement: Supplementary file 1 [file 41419_2026_8896_MOESM3_ESM.pdf]
