# Supplementary material for: YTHDC1 drives senescence evasion in ovarian cancer through m6A-mediated TERT stabilization
Source: Cell Death Dis. 2026 May 30;17(1):953. doi: 10.1038/s41419-026-08896-7 (PMC13748622; doi:10.1038/s41419-026-08896-7)
Supplement: Supplementary file 2 [file 41419_2026_8896_MOESM1_ESM.docx]

**(A)** Representative immunoblot showing YTHDC1 protein levels in the normal ovarian epithelial cell line IOSE-80 and ovarian cancer cell lines (A2780, ES-2, SKOV3, and OVCAR3); Numbers below the blot indicate YTHDC1 band intensity normalized to IOSE-80.

**(B)** Validation of YTHDC1 knockdown efficiency in A2780, ES-2, SKOV3, and OVCAR3 cells using two independent shRNAs.

**(C)** Densitometric quantification of immunoblotting data in Fig. 3E showing relative protein expression of senescence-associated markers and YTHDC1 in A2780 and ES-2 cells following YTHDC1 knockdown. Protein levels were normalized to β-actin and expressed relative to shCtrl. Data are presented as mean ± SD from three independent experiments (n = 3). Statistical significance was assessed by one-way ANOVA with Tukey’s test. *, *P* < 0.05; **, *P* < 0.01; ***, *P* <0.001.

**(D)** Representative flow cytometry histograms showing cell-cycle distribution in A2780 and ES-2 cells after YTHDC1 knockdown.

**(A)** qRT–PCR analysis of YTHDC1 and TERT mRNA levels in OVCAR3 and SKOV3 cells after YTHDC1 knockdown.
**(B)** Representative immunoblots (left) and densitometric quantification (right) of YTHDC1 and TERT protein levels in OVCAR3 and SKOV3 cells following YTHDC1 knockdown. Protein abundance was normalized to β-actin and expressed relative to shCtrl.

Data are presented as mean ± SD from three independent experiments (n = 3). Statistical significance was assessed by one-way ANOVA with Tukey’s test. **, *P* < 0.01; ***, *P* <0.001.

**(A)** TERT promoter luciferase reporter assay in A2780 and ES-2 cells following YTHDC1 knockdown, shown as normalized luciferase activity (relative to shCtrl), *n* = 3.
**(B)** RT–PCR analysis of TERT splice isoforms, showing full-length TERT (TERT-FL) and the truncated TERT-β isoform in A2780 and ES-2 cells with the indicated shRNAs.
**(C)** Validation of cytoplasmic/nuclear fractionation quality by qRT–PCR of GAPDH (cytoplasmic marker) and U6 (nuclear marker) in A2780 and ES-2 cells, *n* = 3.

Data are presented as mean ± SD. Statistical significance was assessed by one-way ANOVA. ns, not significant.

**(A)** Body weight monitoring of ES-2 xenograft-bearing mice treated with vehicle, shYTHDC1 + vehicle, or shYTHDC1 + ABT-263, measured at the indicated time points.

**(B)** Representative H&E staining of liver and kidney sections collected at the study endpoint from the indicated treatment groups (20× and 40×; dashed boxes indicate the regions shown at higher magnification).

Data are presented as mean ± SD. For (A), statistical significance was assessed by two-way ANOVA; ns, not significant.

**(A)** qRT–PCR analysis of YTHDF1, YTHDF2, or YTHDF3 knockdown efficiency and TERT mRNA levels in A2780 and ES-2 cells transfected with control siRNA (siCtrl) or two independent siRNAs targeting each YTHDF gene, n = 3.

**(B)** Representative immunoblots showing TERT protein levels following siRNA-mediated depletion of YTHDF1, YTHDF2, or YTHDF3 in A2780 and ES-2 cells.

Data are presented as mean ± SD. For (A), statistical significance was assessed by one-way ANOVA. ns, not significant. **Table S1. Primer Sequences for Plasmid Construction**

| **Plasmid** | **Primer** | **Sequence** |
| --- | --- | --- |
| **pHIV-YTHDC1-WT** | XbaI-forward | 5’-CGCTGAGTTAACTATTCTAGAATGGCGGCTGA  CAGTCGGGAG-3’ |
|  | BamHI-reverse | 5’-GGGAGGGAGAGGGGCGGATCCTTATCTTCTATA  TCGACCTCTCTCCCC-3’ |
| **pcDNA3.1-YTHDC1-WT** | EcoRI-forward | 5’-TATGCTGGATCTAGAGAATTCATGGCGGCTGAC  AGTCGGGAG-3’ |
|  | HindIII-reverse | 5’-AGTCAGTTAGATATCAAGCTTTTATCTTCTATAT  CGACCTCTCTCCCC -3’ |
| **PLVX-gfp-TERT** | BamHI- forward | 5’-GATGAGCTGTACAAGGGATCCTGCCGCGCGCTC  CCCG-3’ |
|  | XbaI- reverse | 5’-TACCCGGTAGAATTATCTAGATCAGTCCAGGAT  GGTCTTGA-3’ |
| **YTHDC1-W377A** | W377A-sense | 5’-AAAGCGAAGGGTGTAGCGTCCACGCTCCCTG  TA-3’ |
|  | W377A-antisense | 5’-TACAGGGAGCGTGGACGCTACACCCTTCGCTT  TG-3’ |
| **YTHDC1-DM** | W428A-sense | 5’-GGATCTCCTATACACGCGGTGCTTCCAGCAG  GA-3’ |
|  | W428A-antisense | 5’-TCCTGCTGGAAGCACCGCGTGTATAGGAGAT  CC-3’ |
| **Plko.1-shYTHDC1-1** | shYTHDC1-1-sense | 5’-CCGGGAACAGTCCAAGAATGTAGATCTCGAGA  TCTACATTCTTGGACTGTTCTTTTTG-3’ |
|  | shYTHDC1-1-antisense | 5’-AATTCAAAAAGAACAGTCCAAGAATGTAGATC  TCGAGATCTACATTCTTGGACTGTTC-3’ |
| **Plko.1-shYTHDC1-2** | shYTHDC1-2-sense | 5’-CCGGTGCCTCCAGAGAACCTTATAATTCAAGA  GATTATAAGGTTCTCTGGAGGCATTTTTG-3’ |
|  | shYTHDC1-2- antisense | 5’-AATTCAAAAATGCCTCCAGAGAACCTTATAAT  CTCTTGAATTATAAGGTTCTCTGGAGGCA-3’ |
| **Plko.1-shMettl3-1** | shMettl3-1-sense | 5’- CCGGGCCAAGGAACAATCCATTGTTCTCGAGA  ACAATGGATTGTTCCTTGGCTTTTTG-3’ |
|  | shMettl3-1-antisense | 5’- AATTCAAAAAGCCAAGGAACAATCCATTGTT  CTCGAGAACAATGGATTGTTCCTTGGC-3’ |
| **Plko.1-sh Mettl3-2** | shMettl3-2-sense | 5’-CCGGCGTCAGTATCTTGGGCAAGTTCTCGAGAA  CTTGCCCAAGATACTGACGTTTTTG-3’ |
|  | shMettl3-1-antisense | 5’- AATTCAAAAACGTCAGTATCTTGGGCAAG  TTCTCGAGAACTTGCCCAAGATACTGACG-3’ |
| **pGL3-TERT-promote**r | XbaI-F | 5’- GCGTGCTAGCCCGGGCTCGAGGTATTTTTAG  TAGAGACGGGG-3’ |
|  | HindIII -R | 5’-CAGTACCGGAATGCCAAGCTTGGCCAGGGCTTC  CCAC-3’ |

**Table S2. List of Antibodies Used**

| Antibody | Catalog number | Supplier | Ratio for using |
| --- | --- | --- | --- |
| **YTHDC1** | #81504 | Cell Signaling Technology | 1:1000 |
| **YTHDC1** | Ab220159 | Abcam | 1:200 IHC |
| **YTHDC1** | A318 | ABclonal | 1:1000 |
| **Ki67** | #9449 | Cell Signaling Technology | 1:100 |
| **TERT** | ab32020 | Abcam | 1:1000 |
| **P27** | 25614-1-AP | Proteintech | 1:1000 |
| **P53** | Sc-126 | Santa cruz Biotechnology | 1:500 |
| **P21** | #2947 | Cell Signaling Technology | 1:1000 |
| **P16** | #18769 | Cell Signaling Technology | 1:1000 |
| **β-actin** | A3854 | Sigma-Aldrich | 1:40000 |
| **LaminB1** | #13435 | Cell Signaling Technology | 1:1000 |
| **HA** | H9658 | Sigma-Aldrich | 1:10000 |
| **IgG** | #3900 | Cell Signaling Technology |  |
| **γH2AX (Ser139)** | #9718 | Cell Signaling Technology | 1:1000 |
| **METTL3** | #86132 | Cell Signaling Technology | 1:1000 |
| **IL-1α** | 84411-5-RR | Proteintech | 1:500 / 1:50IHC |
| **M6A** | Ab151230 | Abcam | 2μl/10 cm dish |
| **YTHDF1** | HA721302 | Huabio | 1:1000 |
| **YTHDF2** | HA722514 | Huabio | 1:2000 |
| **YTHDF3** | HA724064 | Huabio | 1:5000 |
|  | | | |

**Table S3. Primer Sequences for TRAP Assay**

| **Primers** | **Sequence** |
| --- | --- |
| **TS** | 5’-CY5-AATCCGTCGAGCAGAGTT-3’ |
| **ACX** | 5’-GCGCGGCTTACCCTTACCCTTACCCTAACC-3’ |
| **NT** | 5’-ATCGCTTCTCGGCCTTTT-3’ |
| **TSNT** | 5’-AATCCGTCGAGCAGAGTTAAAAGGCCGAGAAGCGAT-3’ |

**Table S4. Predicted m6A Sites in TERT mRNA**

| **#** | **Position** | **Sequence context** | **Combined score** | **Decision** |
| --- | --- | --- | --- | --- |
| **1** | 655 | GGCCCCCGCCACACGCUAGUGGACCCCGAAGGCGUCUGGGAUGCG | 0.536 | m6A site (Low confidence) |
| **2** | 1032 | UCGCGGCCACCACGUCCCUGGGACACGCCUUGUCCCCCGGUGUAC | 0.577 | m6A site (Moderate confidence) |
| **3** | 1142 | CAGCUCUCUGAGGCCCAGCCUGACUGGCGCUCGGAGGCUCGUGGA | 0.581 | m6A site (Moderate confidence) |
| **4** | 1202 | UUCCAGGCCCUGGAUGCCAGGGACUCCCCGCAGGUUGCCCCGCCU | 0.633 | m6A site (High confidence) |
| **5** | 1404 | GUGGCGGCCCCCGAGGAGGAGGACACAGACCCCCGUCGCCUGGUG | 0.576 | m6A site (Moderate confidence) |
| **6** | 1548 | AACGAACGCCGCUUCCUCAGGAACACCAAGAAGUUCAUCUCCCUG | 0.547 | m6A site (Low confidence) |
| **7** | 1626 | ACGUGGAAGAUGAGCGUGCGGGACUGCGCUUGGCUGCGCAGGAGC | 0.629 | m6A site (High confidence) |
| **8** | 1939 | GGCCCGCCCUGCUGACGUCCAGACUCCGCUUCAUCCCCAAGCCUG | 0.600 | m6A site (High confidence) |
| **9** | 1983 | GACGGGCUGCGGCCGAUUGUGAACAUGGACUACGUCGUGGGAGCC | 0.569 | m6A site (Moderate confidence) |
| **10** | 1989 | CUGCGGCCGAUUGUGAACAUGGACUACGUCGUGGGAGCCAGAACG | 0.703 | m6A site (Very high confidence) |
| **11** | 2181 | GUGCUGCGUGUGCGGGCCCAGGACCCGCCGCCUGAGCUGUACUUU | 0.546 | m6A site (Low confidence) |
| **12** | 2247 | GCGUACGACACCAUCCCCCAGGACAGGCUCACGGAGGUCAUCGCC | 0.662 | m6A site (High confidence) |
| **13** | 2775 | CCUGAGUAUGGCUGCGUGGUGAACUUGCGGAAGACAGUGGUGAAC | 0.590 | m6A site (Moderate confidence) |
| **14** | 2796 | AACUUGCGGAAGACAGUGGUGAACUUCCCUGUAGAAGACGAGGCC | 0.605 | m6A site (High confidence) |
| **15** | 2894 | CGGCCUGCUGCUGGAUACCCGGACCCUGGAGGUGCAGAGCGACUA | 0.591 | m6A site (Moderate confidence) |
| **16** | 2933 | CGACUACUCCAGCUAUGCCCGGACCUCCAUCAGAGCCAGUCUCAC | 0.563 | m6A site (Moderate confidence) |
| **17** | 3326 | CCAAGCAUUCCUGCUCAAGCUGACUCGACACCGUGUCACCUACGU | 0.544 | m6A site (Low confidence) |
| **18** | 3371 | GCCACUCCUGGGGUCACUCAGGACAGCCCAGACGCAGCUGAGUCG | 0.652 | m6A site (High confidence) |
| **19** | 3416 | GAAGCUCCCGGGGACGACGCUGACUGCCCUGGAGGCCGCAGCCAA | 0.635 | m6A site (High confidence) |
| **20** | 3456 | GCCAACCCGGCACUGCCCUCAGACUUCAAGACCAUCCUGGACUGA | 0.649 | m6A site (High confidence) |
| **21** | 3474 | UCAGACUUCAAGACCAUCCUGGACUGAUGGCCACCCGCCCACAGC | 0.736 | m6A site (Very high confidence) |
| **22** | 3885 | CCAGGUGGAGACCCUGAGAAGGACCCUGGGAGCUCUGGGAAUUUG | 0.614 | m6A site (High confidence) |

**Table S5. Sequences of siRNA**

| **Primer** | **Sequence** |
| --- | --- |
| **siYTHDF1-1** | 5’-GACUUUGCUCACUACGAGA(dT)(dT)-3’  5’-UCUCGUAGUGAGCAAAGUC(dT)(dT)-3’ |
| **siYTHDF1-2** | 5’- GGCUGGAGAAUAACGACAA(dT)(dT)-3’  5’- UUGUCGUUAUUCUCCAGCC(dT)(dT)-3’ |
| **siYTHDF2-1** | 5’-GCUCCUGGCAUGAAUACUA(dT)(dT)-3’  5’-UAGUAUUCAUGCCAGGAGC(dT)(dT)-3’ |
| **siYTHDF2-2** | 5’-GACCAAGAAUGGCAUUGCA(dT)(dT)-3’  5’-UGCAAUGCCAUUCUUGGUC(dT)(dT)-3’ |
| **siYTHDF3-1** | 5’-CACCAAUGUCAGAUCCAUA(dT)(dT)-3’  5’-UAUGGAUCUGACAUUGGUG(dT)(dT)-3’ |
| **siYTHDF3-2** | 5’-GGACGUGUGUUUAUAAUUA(dT)(dT)-3’  5’-UAAUUAUAAACACACGUCC(dT)(dT)-3’ |

**Table S6. Primer Sequences for Quantitative RT-PCR**

| **Primer** | **Sequence** |
| --- | --- |
| **YTHDC1** | 5’-AGTGACTCTGGTTCTGAATCTG-3’  5’-CTGGTTTGATCTTTTCGGACAG-3’ |
| **TERT** | 5’-GAGAACAAGCTGTTTGCGGG-3’  5’- AAGTTCACCACGCAGCCATA-3’ |
| **METTL3** | 5’-CATTGCCCACTGATGCTGTG-3’  5’-AGGCTTTCTACCCCATCTTGA-3’ |
| **IL-1α** | 5’-AGAGGAAGAAATCATCAAGC-3’  5’-TTATACTTTGATTGAGGGCG-3’ |
| **IL-6** | 5’-ACTCACCTCTTCAGAACGAATTG-3’  5’-CCATCTTTGGAAGGTTCAGGTTG-3’ |
| **ICAM 1** | 5’-ATGCCCAGACATCTGTGTCC-3’  5’-GGGGTCTCTATGCCCAACAA-3’ |
| **YTHDF1** | 5’-GACCCTCCGATTCCATACCT-3’  5’-TGCTGACCTTGAGACCCACT-3’ |
| **YTHDF2** | 5’-CCAGCCACCATTGCTCCTCC-3’  5’-CCCTTGTTATCCCAAGTTCC-3’ |
| **YTHDF3** | 5’-TAATGCGTATGCTGGTGTCT-3’  5’-ACCTCTTGAGTGTCCCTTGA-3’ |
| **GAPDH** | 5’-AAGTGGTCGTTGAGGGCAATG-3’  5’-ACGGACCAGAGCGAAAGCAT-3’ |
| **ACTB** | 5’-CAGCCTTCCTTCCTGGGCATG-3’  5’-ATTGTGCTGGGTGCCAGGGCAG-3’ |
| **U6** | 5’-CTCGCTTCGGCAGCACA-3’  5’-AACGCTTCACGAATTTGCGT-3’ |
|  |  |

**Table S7. Primer Sequences for Telomere Length qPCR**

| **Primer** | **Sequence** |
| --- | --- |
| **Telg** | 5’-ACACTAAGGTTTGGGTTTGGGTTTGGGTTTGGGTTAGTGT-3’ |
| **Telc** | 5’-TGTTAGGTATCCCTATCCCTATCCCTATCCCTATCCCTAACA-3’ |
| **β-globin F** | 5’-CTTCATCCACGTTCACCTTG-3’ |
| **β-globin R** | 5’-GAGGAGAAGTCTGCCGTT-3’ |

**Table S8. Primer Sequences for RNA Pulldown**

| **Primer** | **Sequence** | |
| --- | --- | --- |
| **T7-TERT-1F** | 5’-GAAATTAATACGACTCACTATAGGGGGCAGCGCCGAGCTGGT-3’ |  |
| **T7-TERT-1R** | 5’-CTGCGCCTACCAGGTGTG-3’ |  |
| **T7-TERT-2F** | 5’-GAAATTAATACGACTCACTATAGGGCAATCGGCCGCAGCCCG-3’ |  |
| **T7-TERT-2R** | 5’-CGTCCAGACTCCGCTTCATC-3’ |  |
| **T7-TERT-3F** | 5’-GAAATTAATACGACTCACTATAGGGACGTGTTCTGGGGTTTGATG-3’ |  |
| **T7-TERT-3R** | 5’-ACAGGCTCACGGAGGTCATC-3’ |  |
| **T7-TERT-4F** | 5’-GAAATTAATACGACTCACTATAGGGATACTCAGGGACACCTCGG-3’ |  |
| **T7-TERT-4R** | 5’-CACGCGAAAACCTTCCTCAG-3’ |  |
| **T7-TERT-5F** | 5’-GAAATTAATACGACTCACTATAGGG GTCCCCGGGAGCTTCCG-3’ |  |
| **T7-TERT-5R** | 5’-GTCACTCAGGACAGCCCAG-3’ |  |
| **T7-TERT-6F** | 5’-GAAATTAATACGACTCACTATAGGG GGAGCACCCCGTAGGGG-3’ |  |
| **T7-TERT-6R** | 5’-TTCTGGAGCTGCTTGGGAAC-3’ |  |
| **T7-LacZ-F** | 5’-GAAATTAATACGACTCACTATAGGGTGTGTAGTCGGTTTATGCAGC-3’ |  |
| **T7-LacZ-R** | 5’-GAGCGGCATTTTCCGTGAC-3’ |  |

**Table S9. Primer Sequences for MS2-TRAP Assay**

| **Primer** | **Sequence** |
| --- | --- |
| **pcDNA3.1-TERT-F** | 5’-TAGCGTTTAAACTTAATGCCGCGCGCTCCCCG-3’ |
| **pcDNA3.1-TERT-1R** | 5’-TCCGAGCTCGGTACCTCACATCCCAGACGCCTTCGG-3’ |
| **pcDNA3.1-TERT-CDS-R** | 5’-TCCGAGCTCGGTACCTCAGTCCAGGATGGTCTTGA-3’ |
| **pcDNA3.1-TERT-CDS-3UTR-R** | 5’-TCCGAGCTCGGTACCCAGTATTTTACTCCCACAGCA-3’ |
| **pcDNA3.1-TERT-2F** | 5’-TAGCGTTTAAACTTAATGCGAACGGGCCTGGAACCA-3’ |
| **pcDNA3.1-TERT-2R** | 5’-TCCGAGCTCGGTACCTCACCCACGACGTAGTCCATGTT-3’ |
| **pcDNA3.1-TERT-3F** | 5’-TAGCGTTTAAACTTAATGAGCCAGAACGTTCCGCAGA-3’ |
| **pcDNA3.1-TERT-3R** | 5’-TCCGAGCTCGGTACCTCATCTGTCAAGGTAGAGACGTGGC-3’ |
| **pcDNA3.1-TERT-4F** | 5’-TAGCGTTTAAACTTAATGCCTCCAGCCGTACATGCG-3’ |
| **pcDNA3.1-TERT-4R** | 5’-TCCGAGCTCGGTACCTCAAGTTCACCACTGTCTTCCGC-3’ |
| **pcDNA3.1-TERT-5F** | 5’-TAGCGTTTAAACTTAATGTCCCTGTAGAAGACGAGGC-3’ |
| **pcDNA3.1-TERT-5R** | 5’-TCCGAGCTCGGTACCGCCATCAGTCCAGGATGGTC-3’ |
| **pcDNA3.1-TERT-6F** | 5’-TAGCGTTTAAACTTAATGTGGCCACCCGCCCACAG-3’ |
| **TERT -PCR1-F** | 5’-TCTTTGTGCTGGTGGCTCC-3’ |
| **TERT -PCR2-F** | 5’-CGTCCAGACTCCGCTTCATC-3’ |
| **TERT -PCR3-F** | 5’-ACAGGCTCACGGAGGTCATC-3’ |
| **TERT -PCR4-F** | 5’-CACGCGAAAACCTTCCTCAG-3’ |
| **TERT -PCR5-F** | 5’-GTCACTCAGGACAGCCCAG-3’ |
| **TERT -PCR1-R** | 5’-CTTCGGGGTCCACTAGCGTG-3’ |
| **TERT -PCR2-R** | 5’-CCCACGACGTAGTCCATGTT-3’ |
| **TERT -PCR3-R** | 5’-TCTGTCAAGGTAGAGACGTGGC-3’ |
| **TERT -PCR4-R** | 5’-AGTTCACCACTGTCTTCCGC-3’ |
| **TERT -PCR5-R** | 5’-GCCATCAGTCCAGGATGGTC-3’ |
| **TERT -PCR6-F** | 5’-GGCTTCCACTCCCCACATAG-3’ |
| **TERT -PCR6-R** | 5’-TGTACAGGGCACACCTTTGG-3’ |
