# Supplementary material for: YTHDC1 drives senescence evasion in ovarian cancer through m6A-mediated TERT stabilization
Source: Cell Death Dis. 2026 May 30;17(1):953. doi: 10.1038/s41419-026-08896-7 (PMC13748622; doi:10.1038/s41419-026-08896-7)
Supplement: Supplementary file 3 [file 41419_2026_8896_MOESM2_ESM.docx]

**Supplementary Material and Methods**

1. **Lentivirus production /transduction and siRNA transfection**

PAX2, pMD2.G, and lentiviral vectors (pHIV, pLVX, and PLKO.1) were co-transfected into HEK293T cells using PEI 40,000 (40816ES02, Yeasen, Shanghai, China). Viral supernatants were harvested 48 and 72 h post-transfection by collecting and centrifuging the cell culture medium. Ovarian cancer cells were transduced with lentiviral supernatant in the presence of 4 μg/mL polybrene. Transduced cells were selected using 1 μg/mL puromycin (60210ES25, Yeasen, Shanghai, China) or 800 μg/mL G418 (11811023, Thermo Fisher Scientific, Waltham, Massachusetts, USA) for one week.

A2780 and ES-2 cells were transfected with siRNA using the Beyotime Lipofectamine kit (C0533, Beyotime, Shanghai, China) according to the manufacturer's instructions. Cells were harvested 48 h post-transfection for RNA and protein extraction for subsequent experiments. The siRNA sequences used are listed in Table S5.

2. **MTT Assay**

A2780, ES-2, SKOV3, and OVCAR3 cells (2,000 cells/well) were seeded into 96-well plates. After adhesion, cells were incubated with MTT solution (HY-15924, MedChemExpress, New Jersey, USA) for 4 h at 37°C. Formazan crystals were dissolved in DMSO and the absorbance was measured at 562 nm after 24, 48, 72, and 96 h.

3. **Colony Formation Assay**

A2780, ES-2, SKOV3, and OVCAR3 cells were seeded at 500, 1500, 1000, and 1000 cells/well in 6-well plates, respectively, and cultured for 9 days. The cells were fixed with 4% paraformaldehyde and stained with 0.1% crystal violet. Colonies containing ≥ 50 cells were visually identified and counted.

4. **Senescence-associated β-galactosidase activity (SA-β-Gal) staining**

Cellular senescence was assessed using a Senescence β-Galactosidase Staining Kit (C0602, Beyotime, Shanghai, China). For cultured cells, samples were fixed, stained overnight at 37°C, washed with PBS, and imaged. For tumor tissues, 5-μm cryosections from OCT-embedded samples were fixed and stained under the same conditions. Following staining, the tissue sections were washed, stained with DAPI, and mounted before observed under a microscope.

5. **Measurement of Intracellular Reactive Oxygen Species (ROS)**

Intracellular ROS levels were quantified using a commercial assay kit (S0033M, Beyotime, Shanghai, China). Briefly, cells were loaded with 10 μM DCFH-DA in serum-free medium for 20 minutes at 37°C. After washing three times to remove the excess probe, cellular fluorescence was analyzed by flow cytometry.

6. **Cell Cycle Analysis**

Cells were harvested by trypsinization, washed with cold PBS, and fixed overnight in cold anhydrous ethanol at -20°C. Following fixation, the cells were treated with RNase A (100 µg/mL) for 30 minutes at 37°C, and then stained with 50 µg/mL propidium iodide (PI; P-4170, Sigma-Aldrich, Michigan, USA) for 10 minutes and their DNA content was analyzed by flow cytometry.

7. **ELISA**

Conditioned medium was collected from YTHDC1-knockdown A2780 and ES-2 cells after 48 hours of culture. The concentrations of IL-1A (EHC176.96, NEOBIOSCIENCE, Shenzhen, China) and IL-6 (EH0001, HUABIO, Hangzhou, China) in the samples were determined using respective ELISA kits according to the manufacturer's protocols, by measuring the optical density (OD) with a microplate reader and calculating the concentrations based on the standard curve equations.

8. **RNA extraction and PCR analyses**

Total RNA was extracted using TRIzol Reagent following the manufacturer’s instructions, and nuclear and cytoplasmic fractions were isolated using the PARIS Kit (AM1921, Thermo Fisher, Waltham, Massachusetts, USA). cDNA was synthesized from 500 ng~1 µg of RNA using the PrimeScript™ RT Reagent Kit (with gDNA Eraser). Alternative splicing of TERT was assessed by semi-quantitative PCR using Dream Taq (K1082, Thermo Fisher, Waltham, Massachusetts, USA) and primers TERT-109-F (5′-GCCTGAGCTGTACTTTGTCAA-3′) and TERT-2531-R (5′-AGGCTGCAGAGCAGCGTGGAGAGG-3′), and the products were resolved by agarose gel electrophoresis. Quantitative RT–PCR was performed with TB Green Premix Ex Taq II (RR820A, Takara Bio, Japan) on a LightCycler 480 (Roche) using primers spanning exon–exon junctions (Table S6); melt-curve analysis confirmed specificity. ACTB was used to normalize total RNA, GAPDH was used for cytoplasmic RNA, and U6 snRNA was used for nuclear RNA. The relative expression was calculated using the 2^–ΔΔCt method from technical triplicates and at least three biological replicates.

9. **Relative Telomere Length Quantification qPCR Assay**

Relative telomere length was measured using a modified quantitative PCR assay as previously described(1, 2). Briefly, 1 × 10^6^ cells from each experimental group were harvested and genomic DNA was isolated using the Biospin Cell Genomic DNA Extraction Kit (BSC05S1, BIOFLUX, Tianjin, China) according to the manufacturer’s instructions. DNA purity and concentration were assessed using a NanoDrop spectrophotometer, and the samples were diluted to 5 ng/μL in nuclease-free water. For each sample, equal amounts of DNA were subjected to two parallel qPCR reactions, using telomere-specific primers (Tel) and single-copy β-globin primers (Table S7). The reactions were performed in triplicate, and the relative telomere length was calculated as the telomere/single-copy gene (T/S) ratio based on the ΔCt method.

10. **Dual-Luciferase reporter assay**

The TERT promoter was PCR-amplified and inserted into the pGL3-Basic vector (primer sequences in Table S1). Cells were co-transfected with the pGL3-TERT promoter and pRL-TK. Firefly and Renilla luciferase activities were measured 24 h after transfection using the Dual-Luciferase Reporter Assay System (E1910, Promega, Madison, Wisconsin, USA). Relative promoter activity was calculated by normalizing the firefly luciferase signal to the Renilla luciferase signal.

11. **m6A RNA immunoprecipitation (MeRIP)**

MeRIP was performed under RNase-free conditions as previously described, with minor modifications (3). Total RNA was extracted using TRIzol reagent and treated with DNase I to remove the genomic DNA. For fragmentation-dependent MeRIP, RNA was chemically fragmented with zinc acetate at 94℃ for 90 seconds to an average size of 100-200 nt, which was confirmed by gel electrophoresis; for the non-fragmented condition, intact RNA was used. In both cases, 1 μg RNA was reserved as input, and 300 μg was incubated with anti-m6A antibody or control IgG in 1x IPP buffer at 4 ℃ for 3 h. The RNA-antibody complex was captured with Dynabeads protein G at 4 ℃ overnight. After sequential washes, the m6A-containing RNA was competitively eluted with 1x IPP buffer containing 1 mg/mL m6A at 37 ℃ for 1 h. The eluted RNA was further enriched by ethanol precipitation in the presence of sodium acetate and a co-precipitant ink (Sigma-Aldrich, Michigan, USA). Purified RNA was reverse-transcribed into cDNA and analyzed by qPCR using TB Green Premix Ex Taq II on a LightCycler 480. Enrichment was quantified relative to IgG and normalized to the input using ΔCt and %input calculations.

12. **RNA pulldown assay**

Biotin-labeled antisense oligonucleotide probes targeting TERT were synthesized using a Ribo™ RNAmax-T7 biotin labeling kit(C11002-1, Ribobio, Guangzhou, Guangdong, China). RNA pulldown was performed with the BersinBioTM Chromatin Isolation by RNA Purification (ChIRP, Bes5104, Bersin Bio, Guangzhou, Guangdong, China) kit according to the literature (4, 5). Briefly, cells were crosslinked, quenched, washed, and freeze-thawed. Cells were lysed in swelling and nuclear lysis buffers with RNase and protease inhibitors, followed by thorough sonication and pre-clearance, with aliquots reserved as input controls. Lysates were mixed with denatured probes and hybridized at 37°C, followed by incubation with streptavidin magnetic beads. The beads were extensively washed and split for RNA and protein analysis. RNA was extracted using TRIzol reagent for qPCR, and protein-bound beads were boiled in loading buffer for western blotting. The primer sequences are listed in Table S8.

13. **MS2-TRAP**

This experiment was performed as methodology described (6). Briefly, TERT fragments were fused with 24 MS2 hairpins and cloned into the pcDNA3.1. These plasmids were co-transfected into A2780 cells along with PMS2-HA-GFP using a Hieff Trans® Liposomal 2000 (40802ES01, Yeasen, Shanghai, China). Forty-eight hours post-transfection, RNA–protein complexes were captured using an RIP assay with an anti-HA antibody. The MS2 hairpins specifically interacted with the PMS2-HA-GFP fusion protein, enabling immunoprecipitation of the PMS2 complex with proteins bound to TERT RNA. Co-purified RNA and proteins were subsequently analyzed using qPCR and western blotting, respectively. Primers used for plasmid construction are listed in Table S9.

14. **Immunofluorescence**

Cells grown on glass coverslips were fixed with 4% paraformaldehyde, permeabilized with 0.3% Triton X-100, and blocked with 10% goat serum. Cells were incubated overnight at 4°C with primary antibodies against YTHDC1 (Ab220159, Rabbit, 1:500, Abcam, Cambridge, UK) , LaminB1 (#13435, Mouse, 1:500, Proteintech, Wuhan, Hubei, China) or γH2AX (#9718, Rabbit, 1:800, Cell Signaling Technology, Massachusetts, USA) followed by Cy3–conjugated secondary antibodies(A-11080, 1:200, Thermo Fisher, Waltham, Massachusetts, USA) or Alexa Fluor 488– conjugated secondary antibodies (A-11005 1:200, Thermo Fisher, Waltham, Massachusetts, USA) for 1 h at room temperature. Nuclei were counterstained with DAPI and images were acquired using a Zeiss LSM 880 confocal microscope.

15. **EdU staining**

Cells were seeded in 96-well plates, and EdU incorporation was performed in the logarithmic growth phase using a commercial kit (C10310, Ribobio, Guangzhou, Guangdong, China). Briefly, the cells were incubated with EdU for 2 h, fixed, permeabilized, reacted with the detection mixture, and counterstained with a nuclear dye. Fluorescence images were acquired using an IX73 microscope (Olympus).

16. **Telomere qFISH**

Telomere qFISH was performed as previously described, with modifications (7). Briefly, cells were cultured to ~80% confluence and treated with 0.1 mg/mL colchicine for 3 h to enrich the mitotic cells. After trypsinization, the cells were swollen in 75 mM KCl for 40 min, fixed in methanol:acetic acid (3:1), and dropped from a height onto pre-cooled slides to spread chromosomes. slides were fixed in 4% formaldehyde, treated with RNase A (100 µg/mL) at 37°C for 1 h, and digested with 0.005% pepsin. After dehydration with graded ethanol, the slides were hybridized with 100 nM Cy3-labeled TelC PNA probe (F1002, PNA Bio, Thousand Oaks, California, USA) in hybridization buffer (70% formamide, 2× SSC, 20 mM Na₂HPO₄, 20 mM Tris-HCl, 0.3% BSA). Denaturation was performed at 85°C for 10 min, followed by overnight incubation in the dark. Post-hybridization washes were carried out at 50°C, and slides were counterstained with DAPI and imaged using a Zeiss LSM 880 confocal microscope. Relative telomere length was quantified from the Cy3 fluorescence intensity using ImageJ software.

17. **Amplification-based single molecule in situ hybridization (asmFISH)**

In situ hybridization of TERT RNA was achieved using the amplification-based single molecule in situ hybridization method as described previously (8), which was performed using the SEERNA® ISH RNA Fluorescence in Situ Detection Kit (SP1011, SEERNA Bioscience, Xiamen, Fujian, China) under the guidance of the standard protocol of the manual. A Cy5-conjugated TERT RNA probe (U21654; Thermo Fisher Scientific, Waltham, Massachusetts, USA) was used. Cells were seeded on slides and permeabilized with 0.1 M HCl for 5 min at room temperature. Hybridization was performed at 37°C sequentially with target probes (4 h), ligation mix (30 min), splint primers in circularization mix (30 min), and rolling circle amplification mix (3 h), with three washes in Wash Buffer between each step. The labelled probes were then hybridized at room temperature for 30 min. Slides were mounted with SlowFade Gold Antifade Mountant containing 0.5 μg/mL DAPI and imaged using a Leica DM6B microscope equipped with a DFC9000GT camera.

18. **In Vivo Tumor Models**

All animal experiments were conducted according to protocols approved by the Animal Care and Use Committee of Xiamen University (animal ethics approval number: XMULAC20250121). All procedures complied with the national and institutional guidelines and regulations aimed at minimizing animal suffering. Animals were housed under standard conditions and humanely euthanized at the end of the study. Six-week-old female BALB/c nude mice were randomized into experimental groups. For subcutaneous xenografts, OVCAR3 cells (1×10⁶) or ES-2 cells (2×10⁶) were injected into the flank. Tumor size was measured every other day starting from day 7 or 9 post-inoculation, and volume was calculated as V = (L × W²)/2. For the abdominal metastasis model, luciferase-labeled A2780 cells with stable YTHDC1 knockdown (1×10⁶) were intraperitoneally injected and bioluminescence was quantified on day 15. For ABT-263 treatment, ES-2 xenograft-bearing mice were administered ABT-263 (50 mg/kg/day; HY-10087, MedChemExpress) by oral gavage for 14 consecutive days, with dosing initiated when tumors were established/palpable (approximately ~100 mm³; day 9 post-implantation). Body weight was recorded every other day throughout the treatment period. At the experimental endpoint, liver and kidney tissues were harvested for H&E staining to assess potential organ toxicity. Group allocation was not blinded. Tumor measurements and bioluminescence quantification were performed using predefined, objective criteria and standardized settings to minimize bias.

19. **Immunohistochemistry**

Paraffin-embedded ovarian cancer tissue sections were obtained from Zhongshan Hospital, Xiamen University, China. This study was approved by the Medical Ethics Committee of Zhongshan Hospital, Xiamen University (ethical approval number 20190003) in accordance with the Helsinki Declaration, and informed consent was obtained from all patients. The sections were dewaxed, rehydrated, and subjected to antigen retrieval in citrate buffer using microwave heating. Immunohistochemical staining was performed using an UltraSensitive SP IHC Kit (KIT-9720, Maxim, Minhou, Fujian, China) following the manufacturer’s instructions. Primary antibodies against YTHDC1 (1:500) and Ki67 (1:100) were used, and sections were incubated overnight at 4°C. After incubation with secondary antibodies, the sections were visualized with diaminobenzidine (DAB, DAB-1031 Maxim, Minhou, Fujian, China) and counterstained with hematoxylin. Finally, the slides were mounted with resin and scanned using the McAudy Motic VM1 scanner. Immunohistochemical staining was scored blindly by two pathologists, and the average score was taken as the final result. The percentage of positive tumor cells was determined in at least five areas and scored as follows: 0, ≤5%; 1, 5–25%; 2, 25–50%; 3, 50–75%; and 4, ≥75%. The intensity of staining in the positive cells was scored as 1 (weak), 2 (moderate), or 3 (intense). The total score was calculated by multiplying the scores for percentage and intensity of staining.

20. **H&E staining**

Liver and kidney tissues harvested from ES-2 subcutaneous tumor-bearing mice at the indicated time were fixed in 4% paraformaldehyde, dehydrated using an automated tissue processor (ASP200S), embedded, and sectioned at 5 μm thickness. Sections were baked at 60 °C for 2 h, followed by staining and mounting on an automated H&E staining and coverslipping system (CHROMAX ST/CV, Leica, Wetzlar, Germany). Images were subsequently acquired by microscopy and whole-slide scanning.

21. **RNA sequencing and bioinformatic analysis**

Total RNA was extracted using TRIzol Reagent and subjected to transcriptome sequencing by the HuaDa Gene Company (Wuhan, Hubei, China) on the MGISEQ-2000 platform (PE150). Raw reads were quality-checked using FastQC, and clean reads were aligned to the human reference genome (GRCh38) using HISAT2. Gene-level counts were obtained using featureCounts and differential expression analysis was performed using DESeq2. Gene set enrichment analysis (GSEA) was conducted in pre-ranked mode using the clusterProfiler (v4.9.0) R package. For RIP-seq and MeRIP-seq analyses, raw reads were filtered using Fastp (v0.23.4) and aligned to the human reference genome (GRCh38) using STAR (v2.7.11b). Normalized coverage files (bigWig) were generated using bamCoverage (deepTools v3.5.0) and visualized with IGV.

**References**

1.	Cawthon RM. Telomere measurement by quantitative PCR. Nucleic Acids Research. 2002;30(10).

2.	Lee M, Hills M, Conomos D, Stutz MD, Dagg RA, Lau LMS, et al. Telomere extension by telomerase and ALT generates variant repeats by mechanistically distinct processes. Nucleic Acids Research. 2014;42(3):1733-46.

3.	Dominissini D, Moshitch-Moshkovitz S, Amariglio N, Rechavi G. Transcriptome-Wide Mapping of N(6)-Methyladenosine by m(6)A-Seq. Methods Enzymol. 2015;560:131-47.

4.	Chu C, Quinn J, Chang HY. Chromatin Isolation by RNA Purification (ChIRP). Jove-J Vis Exp. 2012(61).

5.	Tsuji Y. Optimization of Biotinylated RNA or DNA Pull-Down Assays for Detection of Binding Proteins: Examples of IRP1, IRP2, HuR, AUF1, and Nrf2. International Journal of Molecular Sciences. 2023;24(4).

6.	Liu S, Zhu J, Jiang T, Zhong Y, Tie Y, Wu Y, et al. Identification of lncRNA MEG3 Binding Protein Using MS2-Tagged RNA Affinity Purification and Mass Spectrometry. Appl Biochem Biotechnol. 2015;176(7):1834-45.

7.	Wang Y, Susac L, Feigon J. Structural Biology of Telomerase. Cold Spring Harb Perspect Biol. 2019;11(12).

8.	Lin C, Jiang M, Liu L, Chen XY, Zhao YC, Chen L, et al. Imaging of individual transcripts by amplification-based single-molecule fluorescence

hybridization. New Biotechnol. 2021;61:116-23.
